# Specificity of Herbivore Defense Responses in a Woody Plant, Black Poplar (*Populus nigra*)

**DOI:** 10.1007/s10886-019-01050-y

**Published:** 2019-02-21

**Authors:** Thomas Fabisch, Jonathan Gershenzon, Sybille B. Unsicker

**Affiliations:** Max-Planck-Institute for Chemical Ecology, Hans-Knöll-Strasse 8, 07745 Jena, Germany

**Keywords:** Specificity of plant defense, Salicinoids, Phytohormones, Herbivore-induced volatiles, Trypsin protease inhibitor

## Abstract

The specificity of woody plant defense responses to different attacking herbivores is poorly known. We investigated the responses of black poplar (*Populus nigra*) to leaf feeding by three lepidopteran species (*Lymantria dispar, Laothoe populi* and *Amata mogadorensis*) and two leaf beetle species (*Phratora vulgatissima* and *Chrysomela populi*). Of the direct defenses monitored, increases in trypsin protease inhibitor activity and the salicinoid salicin were triggered by herbivore damage, but this was not herbivore-specific. Moreover, the majority of leaf salicinoid content was present constitutively and not induced by herbivory. On the other hand, volatile emission profiles did vary among herbivore species, especially between coleopterans and lepidopterans. Monoterpenes and sesquiterpenes were induced in damaged and adjacent undamaged leaves, while the emission of green leaf volatiles, aromatic and nitrogen-containing compounds (known to attract herbivore enemies) was restricted to damaged leaves. In conclusion, indirect defenses appear to show more specific responses to attacking herbivores than direct defenses in this woody plant.

**Electronic supplementary material:**

The online version of this article (10.1007/s10886-019-01050-y) contains supplementary material, which is available to authorized users.

## Introduction

Plant chemical defenses of many types are well known to be induced upon attack by insect herbivores. Such induction is sometimes thought to be specifically tailored to the attacking herbivore species giving rise to terms such as the *specificity of plant responses* (Karban and Baldwin 1997), the *specificity of elicitation* (Stout et al. 1998) and the *specificity of induced resistance* (Agrawal 2000). However, the causes and mechanisms of insect herbivore-specific responses in plants are not yet fully understood. Recent studies have investigated whether a plant responds in an herbivore-specific manner may dependent on the feeding guild of the insect, the level of feeding specialization (reviews by Ali and Agrawal 2012; Bonaventure 2014; Heidel-Fischer et al. 2014 and references therein) or salivary cues (Erb et al. 2012) and herbivore-associated microbe communities (Acevedo et al. 2015).

However, most investigations of the specificity of plant response to different insect attackers have focused on only a single defensive compound or compound group. For example, Van Zandt and Agrawal (2004) reported that the volume of pressurized latex, a putative anti-herbivore defense in milkweed, was differentially induced after herbivory when comparing monarchs (*Danaus plexippus)* to swamp milkweed beetles (*Labidomera clivicollis)*. Silva et al. (2017) observed differences in the profiles of tomato volatiles when comparing plants infested by the whitefly *Bemisia tabaci* to plants infested by the leaf miner *Tuta absoluta*. Studies of multiple classes of defenses and defense signals are uncommon.

Research on the specificity of plant defense induction has also concentrated on herbaceous rather than woody plant species, which are less studied due to the methodological problems accompanying their size and longevity (Lämke & Unsicker 2018). However, these characteristics of woody plants may lead to different responses to herbivores. First, throughout their longer lives woody plants may repeatedly encounter the same herbivore species. Second, they may be under constant attack during the growing season, from leaf flush (van Asch and Visser 2007) to senescence (White 2015). Third, among aboveground organs, the larger amount of biomass concentrated in stems may make losses of leaves to herbivores less critical. This might lead to a defense strategy where damaged tissue is sacrificed while defenses are concentrated in surrounding tissue. In light of these possibilities, both specific and non-specific defense responses might be viable strategies for woody plants under different conditions. While non-specific defenses are effective against more herbivores, a specific defense tailored to a single herbivore may be less costly (Onkokesung et al. 2016). However, there are comparatively few studies investigating the specificity of woody plant defense responses after herbivory, such as those conducted in willow (Fields and Orians 2006) and birch (Hartley and Lawton 1987). Yet these studies focus on a narrow set of defensive compounds, while investigations on a broad set of chemical compounds in combination with measurements of defense hormones are still missing.

The aim of this study was to investigate the defense responses of black poplar (*Populus nigra*) towards feeding by five different leaf-chewing insect herbivore species (two coleopterans and three lepidopterans) commonly occurring on poplar. We investigated herbivore-species-specific changes in the defense-related phytohormones salicylic acid (SA) and jasmonic acid (JA), salicinoids, trypsin protease inhibitor activity, and volatile organic compounds in black poplar to obtain a more complete picture about defense responses in woody plants. Salicinoids, a group of phenolic glycosides highly abundant in poplar trees (Boeckler et al. 2011) negatively affect generalist herbivore performance (Hemming and Lindroth 1995; Lindroth and Peterson 1988; Osier and Lindroth 2001). Protease inhibitors (Bradshaw et al. 1990; Haruta et al. 2001) and certain classes of volatiles (Clavijo McCormick et al. 2014b; Unsicker et al. 2015) are also typical poplar compounds reported to be active in defense against herbivores.

Among the herbivores, the two beetle species, *Chrysomela populi* (poplar leaf beetle) and *Phratora vulgatissima* (blue willow leaf beetle), and one of the lepidopteran caterpillar species *Laothoe populi* (poplar hawk moth) used in this study are specialist feeders, according to the classification by Ali and Agrawal (2012), because they feed on only a narrow range of tree species within the Salicaceae. In contrast the lepidopteran caterpillar species *Amata mogadorensis* and *Lymantria dispar* are true generalists, accepting host plant species from different plant families. Most of these herbivores may occur together on *P. nigra,* especially at the end of the season. In this study, we expected to find marked variations in the specificity of poplar defense responses both among various herbivore species and among different classes of defense metabolites. Since defoliation by chewing herbivores typically has only minor effects on the salicinoid concentrations of poplar (Boeckler et al. 2013, Osier and Lindroth 2001), we hypothesized that the induction of these phenolics would be weak and not herbivore species-specific. However, we expected protease inhibitor activity to be differentially induced, especially when comparing lepidopteran with coleopteran herbivores. Differential induction of protease inhibitors between these taxa has been described before (Chung and Felton 2011). It was also reported that the spectrum of volatile organic compounds induced by herbivores depends on feeding mode, the level of feeding specialization (Danner et al. 2018, Rowen and Kaplan 2016) and the composition of their oral secretions (Acevedo et al. 2015). We therefore hypothesized that volatile emission in black poplar would vary depending on the species identity of the attacker. In order to test these hypotheses, we investigated defense responses in both herbivore-damaged and nearby undamaged foliage.

## Material and Methods

### Plants and Insects

*Populus nigra* saplings were grown from cuttings of young trees made in the summer. All genotypes were originally taken from a natural black poplar population located in a floodplain forest on the Oder River of northeastern Germany (52**°**34′1” N, 14**°**38′3″ E). The trees were reared in the greenhouse under summer conditions (24 °C; 60% relative humidity; 16 hr/8 hr, light/dark) in 2-L pots filled with a 1:1 mixture of sand and soil. The experiments were carried out in a controlled environment chamber (20 °C/18 °C, day/night: 60% relative humidity; 16 hr/8 hr, light/dark) to which trees were transferred 24 hr before the start of the experiments. All trees were regularly fertilized and watered once per day.

*Lymantria dispar* caterpillars were hatched from eggs obtained from the US Department of Agriculture (Buzzards Bay, MA, USA), reared on artificial diet (MP Biomedicals LLC, Illkirch, France) in a climate chamber (23 °C, 60% relative humidity, 14 hr/10 hr, light/dark) and used in experiments as 3rd instar larvae. *Laothoe populi* caterpillars were obtained in 1st instar from a commercial provider (The World of Butterflies and Moths, UK, http://www.wobam.co.uk) and reared on black poplar foliage under laboratory conditions until they were used in experiments as 4th instar larvae. *Amata mogadorensis* caterpillars were hatched from eggs obtained from a private breeder (https://www.entomologenportal.de) and reared on black poplar foliage under laboratory conditions until they were used in experiments as 3rd instar larvae. The two beetle species *Chrysomela populi* and *Phratora vulgatissima* were reared from egg clutches collected in old-growth black poplar trees in the field.

### Herbivore Treatments

**Fig. 1:**
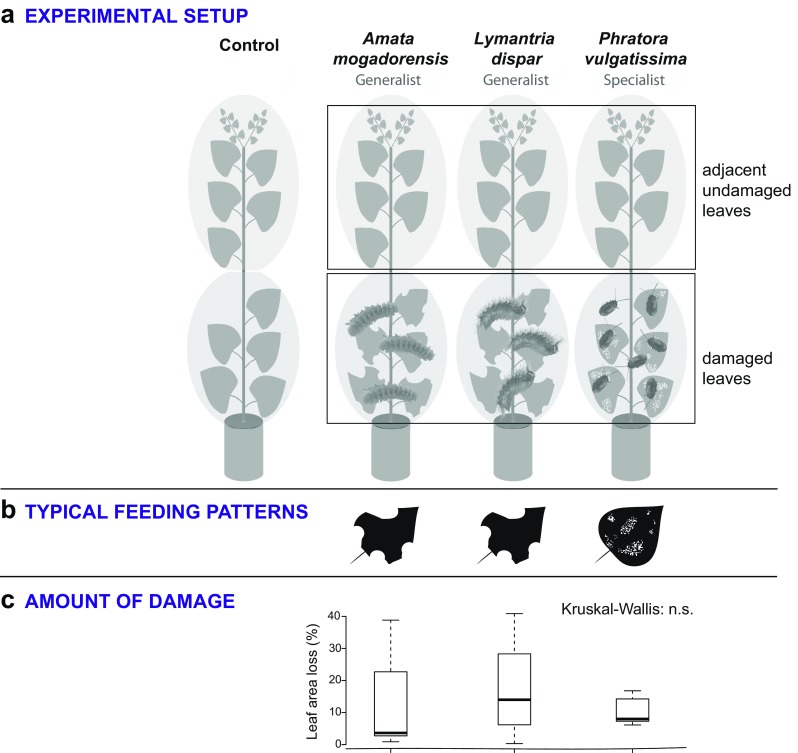
Experimental design (**a**), typical herbivore feeding pattern (**b**) and amount of herbivore damage (**c**) for experiments in which each of three different herbivores was tested on leaves of single black poplar genotypes. The ten full-sized leaves of each sapling were divided into two groups of five leaves each. Each group was wrapped in a polyethylene terephthalate (PET) bag attached to the saplings with cable binders at both ends and supported with a constant flow of charcoal-purified air. The herbivores were caged on the lower leaves and allowed to feed for 44 hr. Lower leaves (from inside the cage) were harvested as “damaged” leaves. Upper leaves from the same sapling were sampled as “adjacent undamaged” leaves. Comparable leaves harvested in the lower and upper leaf pool of non-damaged trees (control) functioned as controls. Differences in the extent of herbivory were analyzed using the non-parametric Kruskal-Wallis test

To study the responses of black poplar to different herbivores, 40 young trees of a single tree genotype were selected. These trees, with a height of approximately 160 cm, were pruned to a height of 80 cm four weeks before the actual experiment started to prevent them from growing too close to the light sources of the climate chamber in which the experiments were conducted. Starting from the pruned site, and counting in basal direction, 10 fully expanded leaves were then split in two sections of five leaves each that were separately enclosed with polyethylene terephthalate (PET) (Toppits Bratschlauch, Minden, Germany) bags fixed at both ends at the poplar stems with cable binders. Bags were left on throughout the duration of the experiment, and charcoal-purified air was pumped into and out of the bags through Teflon tubing at a flow rate of 0.5 L/min to prevent communication between the experimental plants via volatiles. The 40 young trees were split in groups of 10 plants each, resulting in four treatment groups. Three of these four groups received experimental leaf herbivory by one of three different herbivore species, *Lymantria dispar*, *A. mogadorensis* and *P. vulgatissima*. The insects were always released on the lower leaves (designated as “damaged leaves”) with the upper uninfested leaves in the tree designated as “adjacent undamaged leaves” (Fig. 1). One group of 10 trees was not infested with any insects; the lower leaves of these trees served as controls for the damaged leaves, and the upper leaves as controls for the adjacent undamaged leaves. Ten third instar *Lymantria dispar* and *A. mogadorensis* caterpillars or 50 adult *P. vulgatissima* beetles were released on the lower leaves of the different herbivore treatment groups, and allowed to feed for 44 hr. After 24 hr, the number of caterpillars was reduced to six individuals per tree to avoid excessive leaf area loss. Due to space limitations in the controlled environment chamber, the experiment was split into three blocks, each representing an equal number of replicates from each treatment. Thus the first two blocks consisted of three representatives of each treatment and the third block of four replicates of each treatment, resulting in 12, 12 and 16 trees in each block respectively. The time lag between the subsequently processed experimental blocks was 2 days.

### Herbivore Treatment for Volatile Collection

**Fig. 2:**
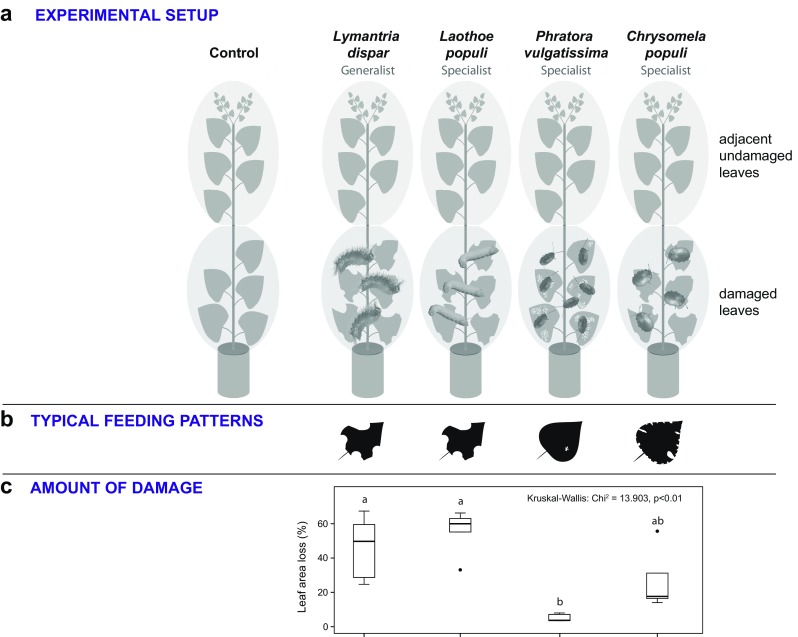
Experimental design (**a**), typical herbivore feeding pattern (**b**) and amount of herbivore damage (**c**) for the experiment testing the effect of four different herbivore species on volatile emission of black poplar saplings of five different genotypes. The approximately ten full-sized leaves of each sapling were divided into two leaf pools containing five leaves each. Each leaf pool was surrounded by polyethylene terephthalate (PET) foil and supported with a constant flow of charcoal-purified air. The lower leaf pool was exposed to four different herbivore treatments, and leaves harvested as described in the Fig. 1 legend. Caterpillars of *Lymantria dispar, Laothoe populi*, and adults of *Phratora vulgatissima* and *Chrysomela populi* were used as herbivores. While *Lymantria dispar, Laothoe populi* and *C. populi* have a biting-chewing feeding mode, *P. vulgatissima* feeds in a piercing-chewing style and has therefore a different feeding pattern. All herbivores were allowed to feed for 44 hr. Differences in the extent of herbivory were analyzed using the non-parametric Kruskal-Wallis test. Pairwise comparisons were made using the *Dunn’s* post hoc test. Black dots represent outliers

To study the emission of volatile organic compounds in response to herbivory, 50 trees of five different tree genotypes were selected. The trees had a height of approximately 80 cm and were younger than the trees in the experiment with a single tree genotype. Therefore, they were not pruned before the experiment but otherwise prepared as described in the previous section. Here, starting from the youngest fully developed leaf, 10 leaves in the basal direction along the stem, were selected to form two leaf pools as described in the previous section. The 50 young trees of five genotypes were evenly split in groups of 10 plants each, resulting in five groups containing 1–3 representatives of each genotype. Four of these groups received experimental leaf herbivory from one of four different herbivore species, *Lymantria dispar*, *Laothoe populi* (poplar hawkmoth)*, P. vulgatissima* and *Chrysomela populi* (poplar leaf beetle). We used a different set of herbivores for this experiment because some of the species were collected in the field and are only available at certain times throughout the year. We have established laboratory cultures for *Lymantria dispar* and *P. vulgatissima* (see above), and thus these species were available for both experiments. The insects were released in the bag enclosing the lower leaves (Fig. 2). One group of trees did not receive any insects and thus functioned as the control group as described above. This experiment was conducted in two consecutive blocks, with 5 replicates of each of the five treatments (*Lymantria dispar*, *Laothoe populi*, *C. populi*, *P. vulgatissima*, non-damaged control) in a block. The time lag between blocks was 2 days. Five 3rd instar caterpillars of *Lymantria dispar* or *Laothoe populi*, 20 adults of *P. vulgatissima* or 5 adults of *C. populi* were released onto the lower leaves and allowed to feed for 44 hr. To avoid complete defoliation the number of individuals in the two caterpillar treatments was reduced to 3 one day after the experiment started.

### Plant Harvest and Quantification of Experimental Leaf Damage

Right after the experiments, all damaged and adjacent undamaged leaves from all treated trees were harvested and photographed after being spread out on a white board with a reference area. After the midribs were removed (due to difficulties in consistently grinding them to a powder), leaves were flash-frozen in liquid nitrogen and then stored in 5 ml plastic vials at −80 °C until further processing. In addition, the equivalent leaves of non-damaged control trees were separately frozen. All leaf material was lyophilized (ALPHA 1–4 LDplus, Christ, Germany) and ground to a fine powder using a paint shaker (Scandex, Pforzheim, Germany) and five stainless steel balls (diameter 3 mm). Experimental leaf area loss in the different herbivore treatments was determined by analyzing the digital images of the leaves with Adobe Photoshop (Version 15.0.0, Adobe Systems Incorporated, San Francisco, USA) following the method described in Boeckler et al. (2013).

### Defense Hormone Analysis

Defense hormones were extracted from an aliquot of 10 mg ground lyophilized leaf material. The aliquot was dissolved in 1 mL of pre-cooled methanol (MeOH) containing the following internal standards [D_6_-abscisic acid (Santa Cruz Biotechnology, Dallas, TX, USA; 40 ng ml^−1^), D_4_-salicylic acid (Santa Cruz Biotechnology; 40 ng ml^−1^), D_6_-jasmonic acid (HPC Standards GmbH, Cunnersdorf, Germany; 40 ng ml^−1^), ^13^C-jasmonoyl-isoleucine (synthesis described in (Kramell et al. 1988), using ^13^C-Ile, Sigma Aldrich; 8 ng ml^−1^)]. The samples were shaken for 30 sec with a paint shaker. Then they were centrifuged at 2000 g for 5 min, and 400 μL of the supernatant were transferred into a new tube. The rest of the supernatant was carefully removed from the solid phase using a pipette. Another 200 μL portion of the supernatant was used for salicinoid analysis. Subsequently, 1 mL of fresh MeOH (without labeled standards) was added to the solid phase before repeating the extraction procedure (shaker + centrifuge). Again, 400 μL (and 200 μL for salicinoids) of the supernatant was collected and combined with the supernatant of the first extraction. The extracts were stored at −20 °C until measurement.

Defense hormones were analyzed using high performance liquid chromatography (Agilent 1100 Varian ELSD, Varian, USA) coupled to a mass spectrometer (API 5000 LC/MS/MS System, AB Sciex, Framingham, MA, USA). The analytes were separated on a C18 column (XDB-C18, 50 × 4.6 mm × 1.8 μm, Agilent, Santa Clara, CA, USA) using a formic acid (0.05% in water) / acetonitrile gradient (flow: 1.1 ml min^−1^) and detected via multiple reactions monitoring (MRM) in negative ionization mode (ion spray at −4500 eV at 700 °C) as described in Vadassery et al. (2012). Data were processed using Analyst 1.5.2 (Applied Biosystems, Foster City, CA, USA), and hormones were quantified relative to the peak area of their corresponding standard.

### Trypsin Protease Inhibitor Activity

Protease inhibitor activity was analyzed via a radial diffusion assay (Jongsma et al. 1993). Samples of 10 mg of freeze-dried leaf material were dissolved in 400 μL of extraction buffer (25 mM Hepes, pH 7.2, adjusted with KOH, 3% PVPP, 2% PVP, 1 mM EDTA). After the addition of one steel ball (diameter 3 mm) and homogenization using a paint shaker (2 × 4 min), the samples were centrifuged at 4 °C and 2000 *g* for 10 min. A 200 μL portion of the supernatant was transferred into a 1 mL centrifuge tube and kept on ice until the analysis. An agar gel (1.8%) was prepared containing 2 μL/mL of fresh trypsin (Merck, Germany) dissolved in 25 mM Hepes-KOH buffer (pH 7.2). After pouring the gel solution onto a square petri dish, the gel was solidified for 3 hr at 4 °C. Subsequently, 5 mm-diameter wells were punched into the gel with a distance of 2 cm to each other using a hollow metal cork-borer. Along with the samples, a standard dilution series of bovine serum albumin (BSA) was added as reference. The gel was then incubated at 4 °C for 22 hr. After the gel was rinsed once with the extraction buffer (Hepes-KOH buffer) containing 10 mM CaCl_2_ and stained with a solution of 72 mg Fast Blue B Salt in 90 mL Hepes buffer (25 mM, pH 7.2, pre-warmed to 37 °C), a 60 mg portion of *N*-acetyl-DL-phenylalanine beta-naphthyl ester (APNE) dissolved in 10 mL N, N-dimethylformamide was added before pouring the solution on the agar plate (pre-warmed to 37 °C as well). Incubation time was 90 min before the staining solution was decanted and the gel was rinsed with water, and a reference curve with BSA was created following the protocol of Bradford (1976) with assays run in triplicate. Before usage, the BSA was reconstituted by mixing with deionized water.

### Salicinoid Analysis

Salicinoids were extracted during the procedure for the extraction of phytohormones (see above) with the addition of 0.8 mg/mL phenyl-β-glucopyranoside as an internal standard. The 2 × 200 μL extracts were combined and 400 μL of milli-Q-purified water was added before measuring the analytes via high performance liquid chromatography (HPLC). Analytes were injected onto a chromatographic column (EC 250 × 4.6 mm NUCLEODUR Sphinx RP, 5 μm, Macherey Nagel, Düren, Germany) connected to a precolumn (C18, 5 μm, 4 × 3 mm, Phenomenex). The temperature of the column oven was set to 25 °C. The mobile phase consisted of two solvents, solvent A (Milli-Q water) and solvent B (acetonitrile), from which solvent B was used in a gradient mode with time/concentration (min/%) of: 0:00/0; 19:00/52; 19:10/100; 21:00/100; 21:10/14; 26:00/14). The flow rate was set to 1 mL/min and injection volume to 20 μL. The signal was detected using photodiode array and evaporative light scattering detectors (Varian, Palo Alto, CA, USA). Using these settings and components, salicin eluted at a retention time of about 5.1 min, salicortin at about 10.2 min and homaloside D at about 15.2 min. The compounds were detected by absorption at 200 nm and identified by comparison of retention time in relation to those of standards isolated from previous work (Boeckler et al. 2013). Quantities were calculated on the basis of peak areas using standard curves prepared with pure standards corrected by the recovery of the internal standard.

### VOC Collection and Analysis

VOCs released from various treatments were collected over a 4 hr period (9:00–13:00 hr) 40–44 hr after the insects were released on basal leaves of treated trees. VOCs in all treatments and leaf pools were trapped on five PDMS (polydimethylsiloxane) tubes (length: 5 mm) attached to 15 cm pieces of acetone cleaned aluminum wire hung inside each bag. PDMS tubes were prepared as described in Kallenbach et al. (2014). After the experiment tubes from each treatment and leaf pool were separately collected in glass vials (VWR International, Darmstadt, Germany) and frozen at −20 °C until further analysis.

Volatile analysis was performed with gas chromatography-mass spectrometry using the Ultra Thermo desorption unit TD20 connected to a quadrupole GC-MS-QP2010Ultra (Shimadzu, Kyoto, Japan). The PDMS tubes were placed in 89 mm glass TD tubes (Supelco, Sigma-Aldrich, Munich, Germany). After desorption in He with a flow rate of 60 mL/min at 200 °C for 8 min, the substances were cyro-focused onto a Tenax® adsorbent trap at −20 °C. The trap was then heated to 230 °C in 10 sec and the sample was injected into an Rtx-5MS column with a length/diameter of 30 m/0.25 mm and a film thickness of 0.25 μm (Restek, Bellefonte, PA, USA). Helium was used as carrier gas with a constant linear velocity of 44.3 cm/s. The TD-GC interface was held at 250 °C. The oven was set to 45 °C for 3 min, raised to 185 °C with an increase of 6 °C/min and subsequently to 320 °C at 100 °C/min with a 15 min hold. Electron impact (EI) mass spectra were recorded at 70 eV in scan mode from 33 to 350 *m*/*z* at a scan speed of 1666 Da/s. The ion source was held at 230 °C. Compounds were identified by comparison of mass spectra and retention times to those of authentic standards and spectra in Wiley and National Institute of Standards and Technology (NIST) libraries.

### Statistical Analyses

Analyses were carried out using SPSS Statistics version 20.0 (IBM, New York, USA). For the volatile analysis using different poplar genotypes, genotypes with more than one replicate were analyzed as one genotypic replicate by taking the mean of the replicates. If necessary the dataset was log-transformed before statistical analysis. To analyze differences in the leaf chemical composition between all treatments, including the control plants, ANOVAs were used. To analyze differences in the leaf chemical composition only between the herbivore treatments ANCOVAs were used with the herbivore damage (leaf area loss) as a co-variable (compound ~ herbivore damage*treatment). Both the ANOVA and ANCOVA models were checked for homoscedasticity, outliers and normal distribution of residuals. For some compounds, the assumptions were violated and could not be rescued with data transformation. Here the treatment was analyzed using the non-parametric Kruskal-Wallis rank sum test. Posthoc comparisons were performed using the *Tukey-Kramer* post hoc test (for ANOVA) and *Dunn’s* post hoc test (for non-parametric Kruskal-Wallis test). For the analysis, the experiment block was left out as well (even if its importance as a factor was significant) because the importance was based on the herbivore damage, which differed between the experiment blocks. Principal component analyses were performed using the online platform MetaboAnalyst (https://www.metaboanalyst.ca). Data were scaled, (mean-centered and divided by the standard deviation of each variable) and transformed using generalized logarithm transformation.

## Results

### Defense Hormones

**Fig. 3:**
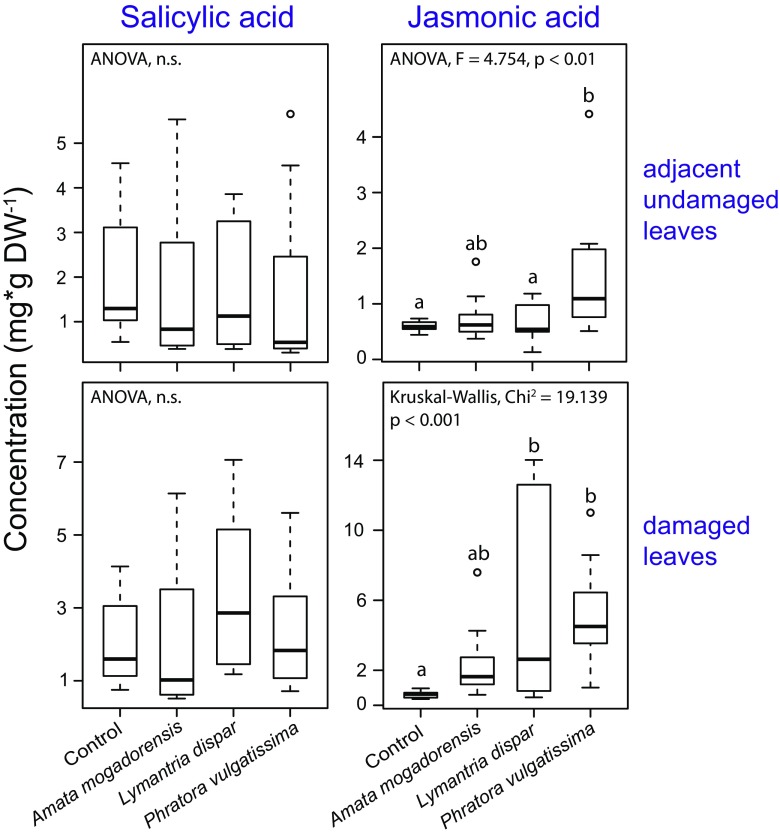
Effect of damage by three herbivore species on the concentrations of two defense hormones, salicylic acid and jasmonic acid, in the damaged and adjacent undamaged leaves of young *Populus nigra* trees as compared to equivalent leaves from non-infested control trees. Samples were collected 44 hr after infestation with caterpillars of *Amata mogadorensis* and *Lymantria dispar*, and adults of the beetle *Phratora vulgatissima*, and from undamaged control plants. The boxplots depict medians ±1.5 x interquartile range of *n* = 10 tree replicates. Pairwise comparisons were conducted using *Tukey’s* post hoc test (ANOVA) and *Dunn’s* post hoc test (Kruskal-Wallis) and are indicated by small letters. Circles indicate outliers. Statistical results comparing only the herbivore treatments are given in Table 1

We assessed the levels of the defense-related phytohormones, salicylic acid (SA) and jasmonic acid (JA), in black poplar leaves from trees of a single genotype after damage by three different herbivore species as compared to leaves from non-infested control trees. Protease inhibitor activity and salicinoids were all measured in samples collected in the same experiment, but volatiles were analyzed in a second, separate experiment. Concerning hormones, SA concentrations in damaged leaves did not differ among the various treatments, including those from non-damaged control trees (Fig. 3). However, JA levels in leaves damaged by *Lymantria dispar* and *P. vulgatissima* were significantly higher than the concentrations in the non-damaged control trees (*Dunn’s* post hoc test: *Lymantria dispar P* = 0.010, *P. vulgatissima P* < 0.001). JA levels in *A. mogadorensis*-infested trees were not significantly different from the control trees, and there were no differences in JA levels among the three different herbivore species (Fig. 3).

In the adjacent undamaged leaves, SA concentrations did not differ between the four treatments (Fig. 3). However, there were significant differences between the four treatments in the JA concentrations of the adjacent undamaged leaves. Pairwise comparisons revealed that JA levels were significantly higher in the *P. vulgatissima*-infested trees (*Tukey-Kramer* post hoc test: *P* = 0.015) compared to the controls and also to the *Lymantria dispar*-infested trees (*Tukey-Kramer* post hoc test: *P* = 0.012). In contrast, the JA levels of *A. mogadorensis*-infested trees were not different from the controls and from the JA levels of the other herbivore-infested trees (Fig. 3). JA concentrations in the adjacent undamaged leaves were generally lower compared to those of damaged leaves.

Insect herbivory measured as % leaf area loss was integrated as a continuous variable in analyses of co-variance (ANCOVA – control trees excluded) to test the effect of leaf damage levels and insect herbivore species identity (main effect) on defense hormone concentrations in the damaged and adjacent undamaged leaves of *P. nigra*. In the damaged leaves SA concentrations were significantly affected by herbivore damage level but there was no significant effect of herbivore species identity (Table 1). JA concentrations in the damaged leaves were significantly affected by both herbivore damage level and herbivore species identity (Table 1).

**Table 1:** Effect of herbivore damage level and herbivore identity on defense metabolites of young *Populus nigra* trees in damaged and adjacent undamaged leaves

	Herbivore damage level				Herbivore identity			
	df	df (error)	F-value	*p* value	df	df (error)	F / Chi^2 (a)^	p value
Damaged leaves								
Phytohormones								
Jasmonic acid	1	26	44.521	**<0.001**	2	26	7.106	**0.003**
Salicylic acid	1	26	13.792	**0.001**	2	26	0.451	0.642
Protease inhibitors								
Trypsin protease								
Inhibitor activity					2	26	4.901^a^	0.086^a^
Salicinoids								
Salicin					2	26	4.880^a^	0.087^a^
Salicortin	1	26	2.043	0.165	2	26	0.42	0.661
Homaloside D	1	26	1.855	0.185	2	26	1.002	0.381
Volatile organic compounds								
Monoterpenoids	1	14	0.157	0.698	3	14	1.883	0.179
Sesquiterpenoids					3	14	8.627^a^	**0.035** ^a^
Aromatic volatiles	1	14	2.356	0.147	3	14	1.567	0.242
Nitrogenous volatiles	1	14	5.177	**0.039**	3	14	5.339	**0.012**
Green leaf volatiles					3	14	1.543^a^	0.672^a^
Adjacent undamaged leaves								
Phytohormones								
Jasmonic acid	1	26	8.816	**0.006**	2	26	8.126	**0.002**
salicylic acid					2	26	0.650^a^	0.722^a^
Protease inhibitors								
Trypsin protease								
Inhibitor activity					2	26	3.582^a^	0.167^a^
Salicinoids								
Salicin					2	26	0.003^a^	0.999^a^
Salicortin					2	26	0.235^a^	0.889^a^
Homaloside D					2	26	0.751a	0.687^a^
Volatile organic compounds								
Monoterpenoids	1	14	2.543	0.133	3	14	2.819	0.077
Sesquiterpenoids	1	14	0.242	0.630	3	14	1.157	0.361
Aromatic volatiles	1	14	2.709	0.122	3	14	2.537	0.099
Nitrogenous volatiles	1	14	0.008	0.929	3	14	0.342	0.796
Green leaf volatiles	1	14	2.195	0.161	3	14	2.173	0.137

Non-parametric Kruskal-Wallis and ANCOVA tests were employed to determine the significance of changes in the concentrations of the phytohormones salicylic acid and jasmonic acid, concentrations of salicinoids, levels of trypsin proteinase inhibitor activity, and emission of major groups of volatiles. The number of replicates was *n* = 10 trees for phytohormones, salicinoids and trypsin protease inhibitor activity and *n* = 5 trees for volatile organic compounds. The tests were performed on the same dataset shown in the graphs, but excluding the control treatment to check for differences only between the plants infested by the different herbivore species. Whenever the assumptions for ANCOVA were met, % leaf area loss (damage) was integrated as a covariate. When ANCOVA assumptions were not met, non-parametric Kruskal-Wallis tests were performed (marked by the letter “a”). Bold numbers indicate significant results

^a^Kruskal-Wallis H-Test

In the adjacent undamaged leaves, JA concentrations were significantly affected by herbivore damage levels and herbivore species identity (Table 1). For SA, herbivore species identity had no effect on the concentration. The effect of herbivore damage levels on SA concentrations in the adjacent undamaged leaves could not be tested as the statistical assumptions for ANCOVA were not met.

### Protease Inhibitor Activity

**Fig. 4:**
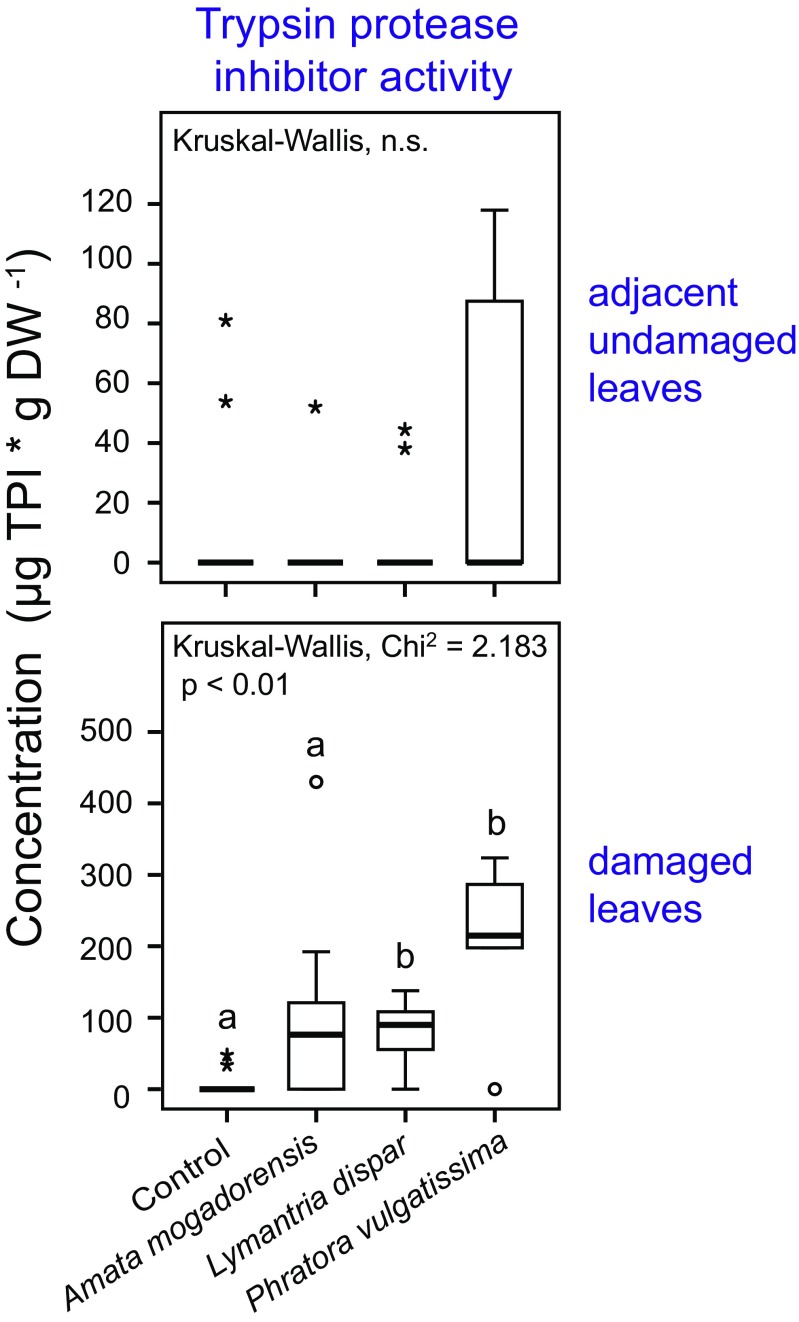
Effect of damage by three herbivore species on the trypsin protease inhibitor activity in the damaged and adjacent undamaged leaves of young *Populus nigra* trees as compared to equivalent leaves from non-infested control trees. Samples were collected 44 hr after infestation with caterpillars of the two lepidopteran species *Amata mogadorensis* and *Lymantria dispar*, adults of the coleopteran species *Phratora vulgatissima*, and untreated control plants. The boxplots represent the median ± 1.5 x interquartile range of n = 10 tree replicates. Pairwise comparisons were conducted using *Dunn’s* post hoc test (Kruskal-Wallis) and are indicated by small letters. Circles indicate outliers and asterisks indicate extreme outliers. The results of statistical analyses comparing only the herbivore treatments are given in Table 1

We found significant differences in the activity of trypsin protease inhibitors (PI) in the damaged leaves of two of the three herbivore treatments as compared to the controls, with the highest activity in the *P. vulgatissima* treatment (*Dunn’s* post hoc test: *P. vulgatissima P* = 0.004, *Lymantria dispar P* = 0.029, Fig. 4). In the adjacent undamaged leaves, there were no significant differences in trypsin PI activity among treatments, but there was a trend for higher activity after *P. vulgatissima* herbivory (Fig. 4). Herbivore species identity did not significantly influence the PI activity in the damaged leaves although a trend was observed. In the adjacent undamaged leaves, trypsin PI activity was not significantly influenced by herbivore species identity (Table 1). The influence of herbivore damage levels on trypsin PI activity could not be tested in either the damaged or adjacent undamaged leaves because assumptions for an ANCOVA were not met.

### Salicinoid Concentrations

**Fig. 5:**
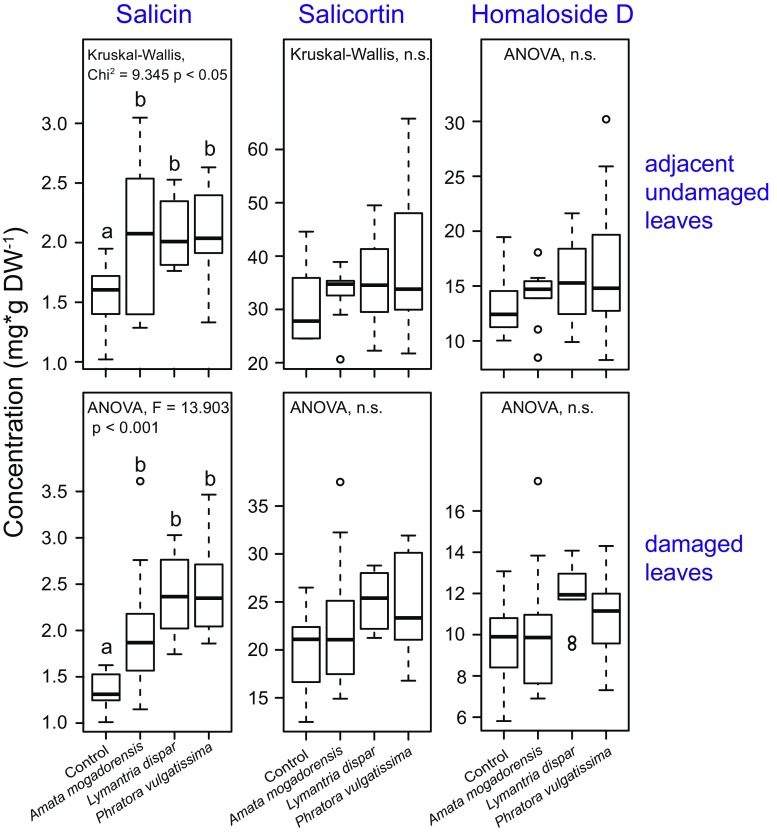
Effect of damage by three herbivore species on the salicinoid concentrations in damaged and adjacent undamaged leaves of young *Populus nigra* trees infested by three different herbivore species as compared to equivalent leaves from non-infested control trees. Samples were collected 44 hr after infestation with caterpillars of two lepidopterans, *Amata mogadorensis* and *Lymantria dispar*, adults of one coleopteran, *Phratora vulgatissima*, and undamaged control plants. The box plots represent median ± 1.5 x interquartile range for n = 10 tree replicates. Pairwise comparisons were conducted using *Tukey’s* post hoc test (ANOVA) and *Dunn’s* post hoc test (Kruskal-Wallis) and are indicated by small letters. Circles indicate outliers. Statistical results comparing only the herbivore treatments are given in Table 1

Three different salicinoids, salicin, salicortin and homaloside D, were detected in black poplar leaves in this study. In the damaged leaves, we found significant differences in the salicin levels of the herbivore-infested trees as compared to the non-infested control trees (Fig. 5). Pairwise comparisons revealed significant inductions by all herbivore species versus the uninfested control trees (*Tukey-Kramer* post hoc test: *A. mogadorensis P* = 0.010, *Lymantria dispar P* < 0.001, *P. vulgatissima* P < 0.001), but no differences among the herbivore treatments were observed. Furthermore, we found no significant differences among treatments for salicortin and homaloside D (Fig. 5).

In the adjacent undamaged leaves, salicin levels were also significantly different in all herbivore-infested trees when compared to non-damaged control trees (*Dunn’s* post hoc test: *A. mogadorensis P* = 0.024, *Lymantria dispar P* = 0.008, *P. vulgatissima P* = 0.012), and there were no significant differences among the herbivore treatments. The levels of salicortin and homaloside D were not significantly different when all treatments were compared (Fig. 5).

In the damaged leaves, herbivore identity did not significantly affect the concentration of salicin although a trend was observed. However, the influence of herbivore damage levels on salicin concentration could not be tested because ANCOVA assumptions were not met. Salicortin and homaloside D levels were not affected by herbivore damage levels or by herbivore identity (Table 1). Also, in the adjacent undamaged leaves the influence of herbivore damage level could not be tested as statistical assumptions were not met. However, herbivore species identity did not significantly affect the concentrations of the three salicinoids measured. (Table 1).

### Volatile Organic Compounds

To determine if different herbivore species cause different volatile responses in black poplar we set up a second experiment using multiple black poplar genotypes and a somewhat different set of herbivore species (Fig. 2). Phytohormone patterns in response to this set of insect herbivores were similar to the patterns observed in the first experiment (Fig. 3, Fig. S2). Altogether 86 volatile organic compounds were measured in this experiment, of which 69 could be (tentatively) identified (Table S1). A PCA performed with all identified volatiles measured in the headspace of the different treatments showed some separations between the herbivore treatments and the control treatment (Fig. S3). The volatile blends were further classified as monoterpenoids, sesquiterpenoids, green leaf volatiles (GLVs), aromatic compounds, nitrogenous compounds and “other volatiles” (compounds that did not fall into any of the chemical classes listed above), as we know from previous studies that certain volatile groups such as GLVs and nitrogenous compounds play essential roles in direct and indirect poplar defense.

**Fig. 6:**
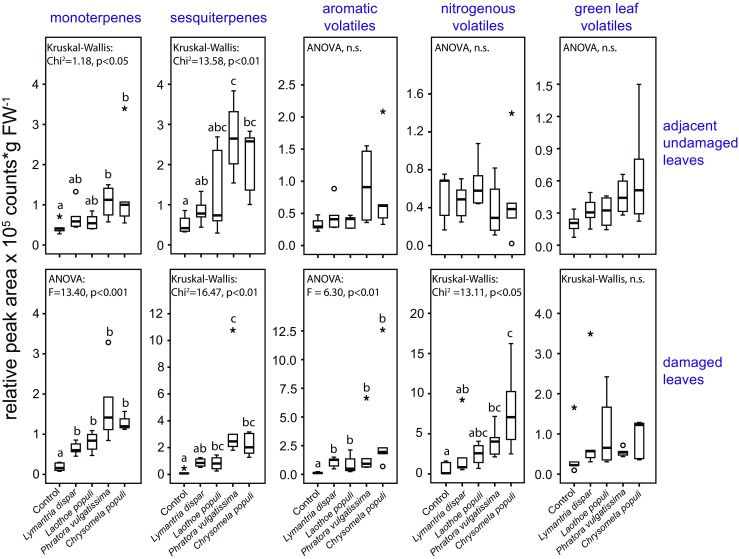
Effect of damage by four herbivore species on the relative amounts of major groups of volatile organic compounds emitted from damaged (lower row) and adjacent undamaged leaves (upper row) of young *Populus nigra* trees as compared to equivalent leaves from non-infested control trees. Samples were collected 44 hr after infestation with caterpillars of two lepidopteran species, *Lymantria dispar* and *Laothoe populi*, adults of two coleopteran species, *Phratora vulgatissima* and *Chrysomela populi*, and untreated control plants. The box plots represent median ± 1.5 x interquartile range for *n* = 5 tree replicates. Pairwise comparisons were conducted using the *Tukey-Kramer* post hoc test (ANOVA) and *Dunn’s* post hoc test (Kruskal-Wallis) and are indicated by small letters. Circles indicate outliers and asterisks indicate extreme outliers. Statistical results comparing only the herbivore treatments are given in Table 1

Monoterpene emission from damaged leaves was significantly higher compared to emission from equivalent leaves on control trees (Fig. 6 lower row, *Tukey-Kramer* post hoc test: *Lymantria dispar P* = 0.008, *Laothoe populi P* < 0.001, *P. vulgatissima* P < 0.001, *C. populi* P < 0.001) but there were no significant differences found among the different herbivore treatments (Fig. 6 lower row). We also observed significant increases in the emission of sesquiterpenes when comparing non-infested control trees with herbivore-infested trees, except for the *Lymantria dispar* infested trees (Fig. 6 lower row, *Dunn’s* post hoc test: *Laothoe populi P* = 0.044, *P. vulgatissima* P < 0.001, *C. populi P* = 0.001). Additionally, significant differences in sesquiterpene emissions were found between *Lymantria dispar-* and *P. vulgatissima*-infested trees with the beetle *P. vulgatissima* inducing higher levels (*Dunn’s* post hoc test: *P* = 0.036). A similar trend was observed between *Lymantria dispar-* and *C. populi*-infested trees (*Dunn’s* post hoc test: *P* = 0.086). The emission of aromatic volatiles in the damaged leaves of all herbivore-infested trees was significantly increased compared to equivalent leaves on the control trees (Fig. 6 lower row, *Tukey-Kramer* post hoc test: *Lymantria dispar P* = 0.026, *Laothoe populi P* = 0.049, *P. vulgatissima P* = 0.011, *C. populi* P = 0.001), but no significant differences were observed among the different herbivore treatments. The emission of nitrogenous volatiles from damaged leaves was significantly increased in the beetle-infested trees (Fig. 6 lower row, *Dunn’s* post hoc test: *P. vulgatissima P* = 0.012, *C. populi* P = 0.001). Their emission was not significantly different between the control trees and the trees infested by the two lepidopteran species *Lymantria dispar* and *Laothoe populi*. There were also significant differences in the emission of nitrogenous volatiles between trees infested by *Lymantria dispar* and trees infested by *C. populi* (Fig. 6 lower row, *Dunn’s* post hoc test: *P* = 0.028). There were no significant differences in GLV emission from damaged leaves between the non-infested controls and any of the different herbivore treatments (Fig. 6 lower row). There were marginally significant differences in the damaged leaves with respect to the emission of “other volatiles”, but posthoc comparisons did not show any significant differences among the treatments (Fig. S1).

From the adjacent undamaged leaves, monoterpene emission differed significantly among the treatments (Fig. 6, upper row). While the two caterpillar species (*Lymantria dispar* and *Laothoe populi*) did not significantly induce monoterpene emission as compared to the equivalent leaves on non-damaged control trees, the two beetle species did (*Dunn’s* post hoc test: *P. vulgatissima P* = 0.008, *C. populi P* = 0.006). Trends towards differences in monoterpene emission were also observed between *Laothoe populi-* and both beetle-infested trees (*Dunn’s* post hoc test: *P. vulgatissima P* = 0.062, *C. populi P* = 0.060). Sesquiterpene emission in the adjacent undamaged leaves differed significantly between trees infested by beetles in the basal leaves and equivalent leaves on control trees (*Dunn’s* post hoc test: *P. vulgatissima P* = 0.002, *C. populi P* = 0.005). In contrast, the two caterpillar species did not significantly induce sesquiterpene emission from undamaged leaves (Fig. 6, upper row). Differences in sesquiterpene emission were also observed between *Lymantria dispar-* and *P. vulgatissima-*infested trees (*Dunn’s* post hoc test: *P* = 0.032). For aromatic and nitrogenous volatiles as well as for green leaf volatiles and other volatiles there were no significant differences among the treatments (Fig. 6 upper row, Fig. S1).

In the damaged leaves the emission of nitrogenous volatiles was significantly affected by the herbivore damage level and herbivore species identity, while sesquiterpene emission from damaged leaves was influenced by herbivore species identity (Table 1). In adjacent undamaged leaves none of the classified volatile groups was significantly affected by herbivore damage level and herbivore identity.

## Discussion

In this study we found that young black poplar trees damaged by the three different leaf-chewing herbivores tested in the single genotype experiment showed increases in the defense hormone jasmonic acid (JA), the salicinoid salicin and trypsin protease inhibitor activity. This was mainly observed in the damaged foliage, but in case of JA, also in the adjacent undamaged foliage. Additionally, all four herbivores tested in the second experiment induced different volatile organic compounds in the damaged as well as the adjacent undamaged foliage. While there was no herbivore-species-specificity for elicitation of the direct defenses surveyed, black poplar did display herbivore-specific emission of several classes of volatiles, in particular sesquiterpenes and nitrogenous compounds. In the case of sesquiterpenes the specificity of elicitation was also visible systemically in undamaged foliage adjacent to the attacked leaves.

When analyzing the two major defense-related phytohormones JA and SA, we found JA to be induced by the two leaf-chewing herbivores *Lymantria dispar* and *P. vulgatissima,* but not by *A. mogadorensis*. (Figure 3, Fig. S2). Local JA induction upon herbivore damage is a common phenomenon in herbaceous and woody plant species (Erb et al. 2012; Singh et al. 2016; Irmisch et al. 2014). The fact that SA was not induced by most of the herbivores investigated is in agreement with the literature. It is well documented that SA is mainly triggered by piercing-sucking insects like aphids (Li et al. 2016; Thaler et al. 2012) or infections by biotrophic pathogens (Kunkel and Brooks 2002). The general lack of SA induction by most of the herbivore species tested and induction of JA suggest a lack of specificity of defense signaling. The only exception was the specialist *Laothoe populi* that triggered the induction of SA in damaged leaves (Fig. S2). We also found that SA levels in damaged leaves were significantly affected by the amount of herbivore damage inflicted (ANCOVA, Table 1), even though there were no differences in SA concentrations between the different herbivore treatments (Fig. 3, Fig. S2). This result differs from other studies, where SA was not significantly influenced by chewing herbivores (Kawazu et al. 2012; Niveyro et al. 2013; Soler et al. 2012) although increasing and decreasing concentrations are also reported (Agrawal et al. 2014; Diezel et al. 2009). These observations demonstrate the complexity of the perception network involved in the recognition of herbivores by plants. This probably involves not only salivary cues, regurgitants and feces of herbivores, but also the associated herbivore microbiota. Investigations about the interaction of plants, herbivores and herbivore-associated microbes are just beginning and general models are hard to establish (Acevedo et al. 2015). The results obtained here and in other studies show that SA levels do respond to herbivory in a more subtle way than usually appreciated. The effects of the resulting signaling processes on the deployment of defenses are not known. Specificity might also be revealed by measurements of other hormones, such as ABA, ethylene and cytokinins (Erb et al. 2012), which were not quantified here.

Feeding by the generalist caterpillar species *Lymantria dispar* and one specialized leaf beetle, *P. vulgatissima*, increased the activity of trypsin protease inhibitors in damaged leaves (Table 1, Fig. 4). Also *A. mogadorensis* visibly increased the activity, although the differences were non-significant. The increased activity of protease inhibitors after wounding is a well-known inducible defense mechanism of plants (Jongsma and Bolter 1997). Since the production of protease inhibitors is associated with significant fitness costs (Zavala et al. 2004), their formation only in response to damage rather than being constitutively produced is understandable. Green and Ryan (1972) found the induction of protease inhibitors to be dependent on the number of wounding sites and the time after wounding. Although there were no significant differences in trypsin protease inhibitor activity in the leaves damaged by the different herbivore species, we observed a trend towards differential inductions (Table 1), which was probably caused by the higher numbers of wound sites from *P. vulgatissima* herbivory.

In contrast to most other black poplar metabolites measured, the major salicinoids, salicortin and homaloside D, were not induced by any of the herbivore species. A significant induction by leaf chewing caterpillars and beetles was only observed in the case of salicin (Fig. 5). Although there is little doubt about the role of salicinoids as defense compounds of Salicaceae plants (Boeckler et al. 2011), their induction patterns after herbivore attack are highly variable. While inductions of salicinoids are evident in some studies (Clausen et al. 1989; Fields and Orians 2006; Rubert-Nason et al. 2015; Stevens and Lindroth 2005) this is not always the case (Boeckler et al. 2013). The variability of herbivore-triggered salicinoid induction may arise because the levels of these phenolic compounds are influenced by many other factors. The most prominent factor is the genotype, which has been observed in many studies to cause much larger variation in salicinoid concentration than defoliation by herbivores (Osier and Lindroth 2001; Rubert-Nason et al. 2015). Other factors are the availability of nutrients and water (Hale et al. 2005) as well as organ, developmental and seasonal variation (Boeckler et al. 2011). Furthermore, individual salicinoids may be differentially induced after herbivory. The lower concentration of salicin compared to the other salicinoids measured does not necessarily mean that its defensive role is less important (Boeckler et al. 2016). In other species, inducible anti-herbivore metabolites with comparatively low concentrations but high impact on herbivores are known, such as indolic glucosinolates (Jeschke et al. 2016; Tian et al. 2005). Future studies should aim to investigate the toxicity and deterrency of herbivore-inducible salicin in comparison to the other less-inducible salicinoids.

When volatiles were measured, herbivory by two lepidopteran species and two leaf beetle species led to significant inductions of almost all major volatile groups (Fig. 6). The inducibility of plant volatiles after herbivory has been shown in both herbaceous (e.g. Fontana et al. 2009; Kigathi et al. 2013; Piesik et al. 2016; Skoczek et al. 2017) and woody plants (e.g. Courtois et al. 2016; Giacomuzzi et al. 2017; Maja et al. 2015) including poplar trees (Clavijo McCormick et al. 2014a; Philippe and Bohlmann 2007). In black poplar, nitrogenous volatiles released upon herbivory have been the focus of attention because they play a major role in attracting natural enemies of herbivores (Clavijo McCormick et al. 2014a). In other plant systems, terpenoids and GLVs are well-known to be involved in the attraction of natural enemies of herbivores (Turlings and Erb 2018). The induction of most of the groups of black poplar volatiles measured has been reported to be associated with JA signaling (Luck et al. 2016; Martin et al. 2003; Semiz et al. 2012). Herbivore-induced increases in protease inhibitor activity have also been connected with elevated jasmonate levels (Haruta et al. 2001; Lomate and Hivrale 2012). These reports are consistent with the JA induction measured in this study where we showed that an assortment of leaf-chewing herbivores all trigger increases in JA.

Elevated JA levels were found both in herbivore damaged leaves and in adjacent undamaged leaves (Fig. 3, Fig. S2) and the effect in adjacent undamaged leaves was dependent on the identity of the attacking herbivore species (Table 1). The systemic induction of JA in adjacent undamaged leaves after herbivory is a known phenomenon in herbs (Singh et al. 2016), but woody plants such as poplar have not always given consistent results. While herbivory by *Lymantria dispar* caused JA inductions exclusively in damaged poplar leaves (Clavijo McCormick et al. 2014b), other studies found JA also increased in the adjacent undamaged leaves (Babst et al. 2009; Boeckler et al. 2013). In the present study, *Lymantria dispar* feeding also led to significantly increased JA levels only in damaged leaves. A trend for higher JA levels in damaged leaves was also visible after feeding by the other generalist caterpillar species, *A. mogadorensis*. However, feeding by the beetle *P. vulgatissima* resulted in significantly higher amounts of JA in both the damaged and adjacent undamaged leaves (Fig. 3). We also observed significant systemic induction of salicin in the adjacent undamaged leaves of black poplar and of monoterpenes and sesquiterpenes as has been reported previously for this species (Clavijo McCormick et al. 2014b; Unsicker et al. 2015). In contrast the most prominent compounds induced only in herbivore-damaged leaves were the trypsin protease inhibitors and the nitrogen-containing volatiles (Fig. 4, Fig. 6). In herbaceous plants, herbivory commonly increases protease inhibitor activity significantly in both damaged and adjacent undamaged leaves (Arce et al. 2017; Bozorov et al. 2017; Lomate and Hivrale 2012). This is not true for poplar where induction in adjacent undamaged leaves (Bradshaw et al. 1990) has been reported to be much weaker and delayed compared to the induction in herbivore-damaged leaves (Haruta et al. 2001). Nitrogen-containing volatiles have previously been reported to be emitted only from herbivore-damaged foliage of black poplar and not systemically (Clavijo McCormick et al. 2014a; Unsicker et al. 2015). This may explain their use by herbivore predators and parasitoids as reliable cues to locate prey and hosts (Clavijo McCormick et al. 2014b).

The volatile bouquets released from black poplar upon herbivore damage differed between the lepidopteran and coleopteran species used in this experiment (Fig. 6, Fig. S3), especially for terpenoids, which were more abundant after coleopteran damage. Similar emission profiles of black poplar have been shown previously (Clavijo McCormick et al. 2014a, 2014b; Unsicker et al. 2015), even though a different volatile collection method was used here. In herbaceous plants, the emission of specific volatile patterns by different herbivore species is known (Cai et al. 2014; Danner et al. 2018; Hare and Sun 2011; Pinto-Zevallos et al. 2018; Turlings et al. 1998) and the pattern of stronger volatile induction after beetle herbivory was also observed (Hare and Sun 2011). At least one other woody plant also showed stronger induction of terpene emission after attack by coleopteran compared to lepidopteran herbivores (Moreira et al. 2013). Several possibilities might be responsible for herbivore species-specific defense responses in plants, including the type of damage and presence of specific elicitors (Ali and Agrawal 2012; Cai et al. 2014; Dicke et al. 2009; Rowen and Kaplan 2016). Specialist herbivores are thought to induce more total volatiles than generalists, although these patterns are not the same for each chemical class (Rowen and Kaplan 2016). One of the herbivore species employed in the present study can be classified as a generalist (*Lymantria dispar*) and the other three are specialists (*Laothoe populi, P. vulgatissima, C. populi*). However, the volatile pattern observed after herbivory differed more based on taxonomic grounds between lepidopterans and coleopterans than based on the degree of specialization.

In summary, our investigation demonstrated that both direct and indirect defenses are induced in black popular by a range of different herbivores. However, the induction of protease inhibitor activity (only in damaged leaves) and salicin (in both damaged and adjacent undamaged leaves) is not specific to the attacking herbivore species. Moreover, the bulk of salicinoids are constitutively present and do not change in concentration with attack. In contrast, the induced volatiles, of which some are known to play a role in indirect defense, do show specific responses to herbivores. The emission pattern from damaged and adjacent undamaged leaves differs between lepidopteran and coleopteran herbivores. Whether this pattern is characteristic of other woody plants requires further investigation.

## Electronic supplementary material

ESM 1 (DOCX 1.89 MB)
