# Supplementary material for: Specificity of Herbivore Defense Responses in a Woody Plant, Black Poplar (Populus nigra)
Source: J Chem Ecol. 2019 Feb 21;45(2):162–77. doi: 10.1007/s10886-019-01050-y (PMC6469625; doi:10.1007/s10886-019-01050-y)
Supplement: Supplementary file 1 — (DOCX 1.89 MB) [file 10886_2019_1050_MOESM1_ESM.docx]

SPECIFICITY OF HERBIVORE DEFENSE RESPONSES IN A WOODY PLANT, BLACK POPLAR (*POPULUS NIGRA*)

Supplemental data

THOMAS FABISCH; JONATHAN GERSHENZON & SYBILLE B. UNSICKER*

*Max-Planck-Institute for Chemical Ecology, Hans-Knöll-Strasse 8, 07745 Jena/Germany*

**Corresponding author: Phone: +49 (0) 3641 571328,*

[*sunsicker@ice.mpg.de*](mailto:sunsicker@ice.mpg.de)

*orcid.org/0000-0002-9738-0075*


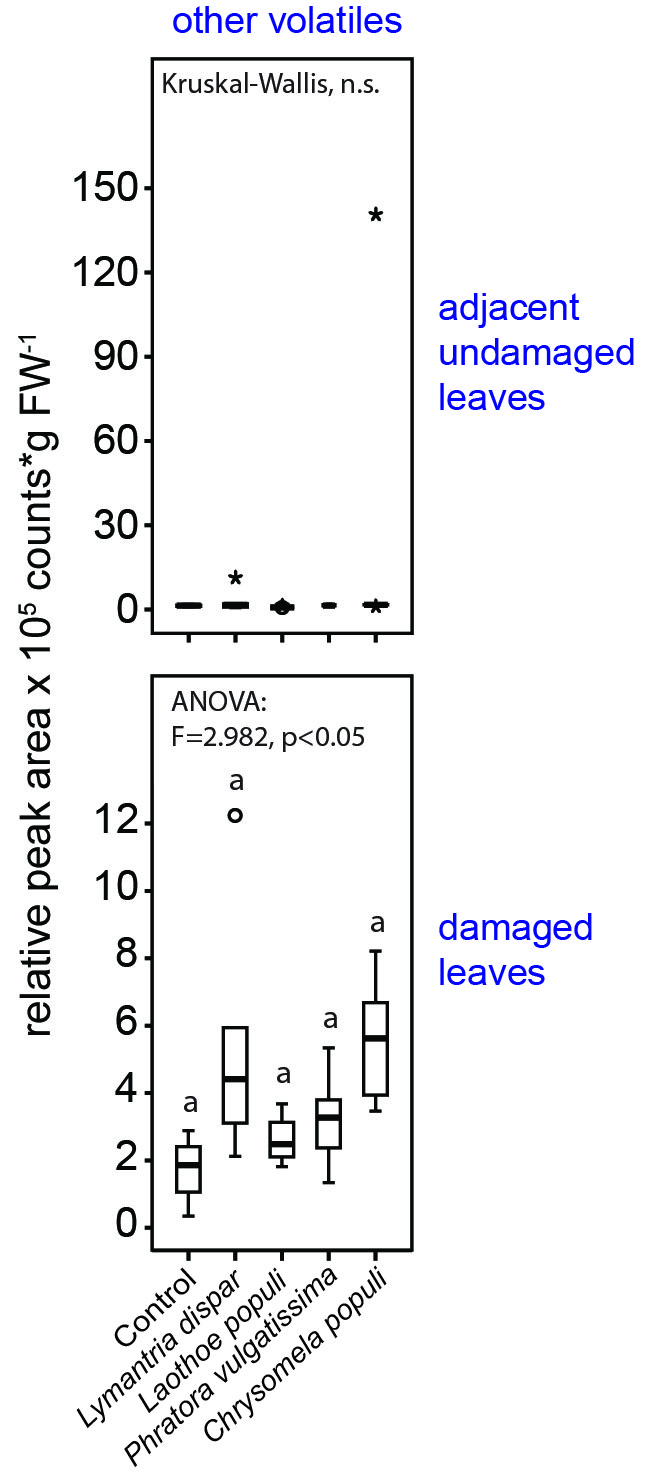


**Fig S1** Effect of damage by four herbivore species on the relative amounts of the volatile group “others” emitted from damaged and adjacent undamaged leaves of young *Populus nigra* trees as compared to equivalent leaves from non-infested control trees.

Samples were collected 44 h after infestation with caterpillars of two lepidopteran species, *Lymantria dispar* and *Laothoe populi*, adults of two coleopteran species, *Phratora vulgatissima* and *Chrysomela populi*, and from untreated control plants. The box plots represent median ± 1.5 x interquartile range for n=5 tree replicates. Letters indicate the results of *Tukey-Kramer* (ANOVA) and *Dunn’s post hoc testing* (Kruskal-Wallis). Circles indicate outliers and asterisks indicate extreme outliers

**
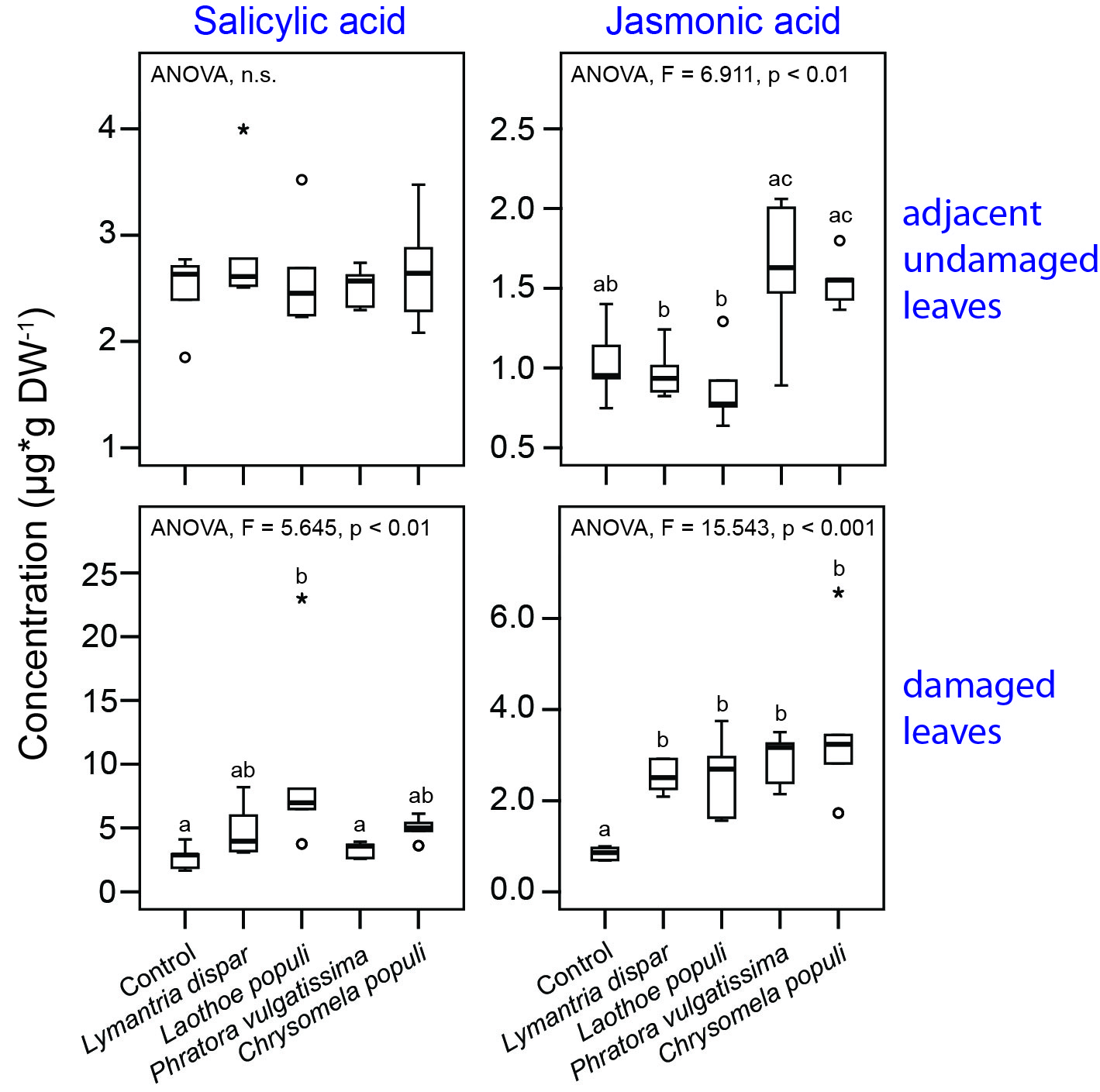
**

**Fig S2** Effect of damage by four herbivore species on the concentrations of the two defense-related phytohormones, jasmonic acid and salicylic acid, in damaged and adjacent undamaged leaves of young *Populus nigra* trees as compared to equivalent leaves from non-infested control trees.

Samples were collected 44 h after infestation with caterpillars of two lepidopteran species, *Lymantria dispar* and *Laothoe populi*, adults of two coleopteran species, *Phratora vulgatissima* and *Chrysomela populi*, and untreated control plants. The box plots represent median ± 1.5 x interquartile range for n=5 tree replicates. Letters indicate the results of *Tukey-Kramer* (ANOVA) and *Dunn’s post hoc testing* (Kruskal-Wallis). Circles indicate outliers and asterisks indicate extreme outliers


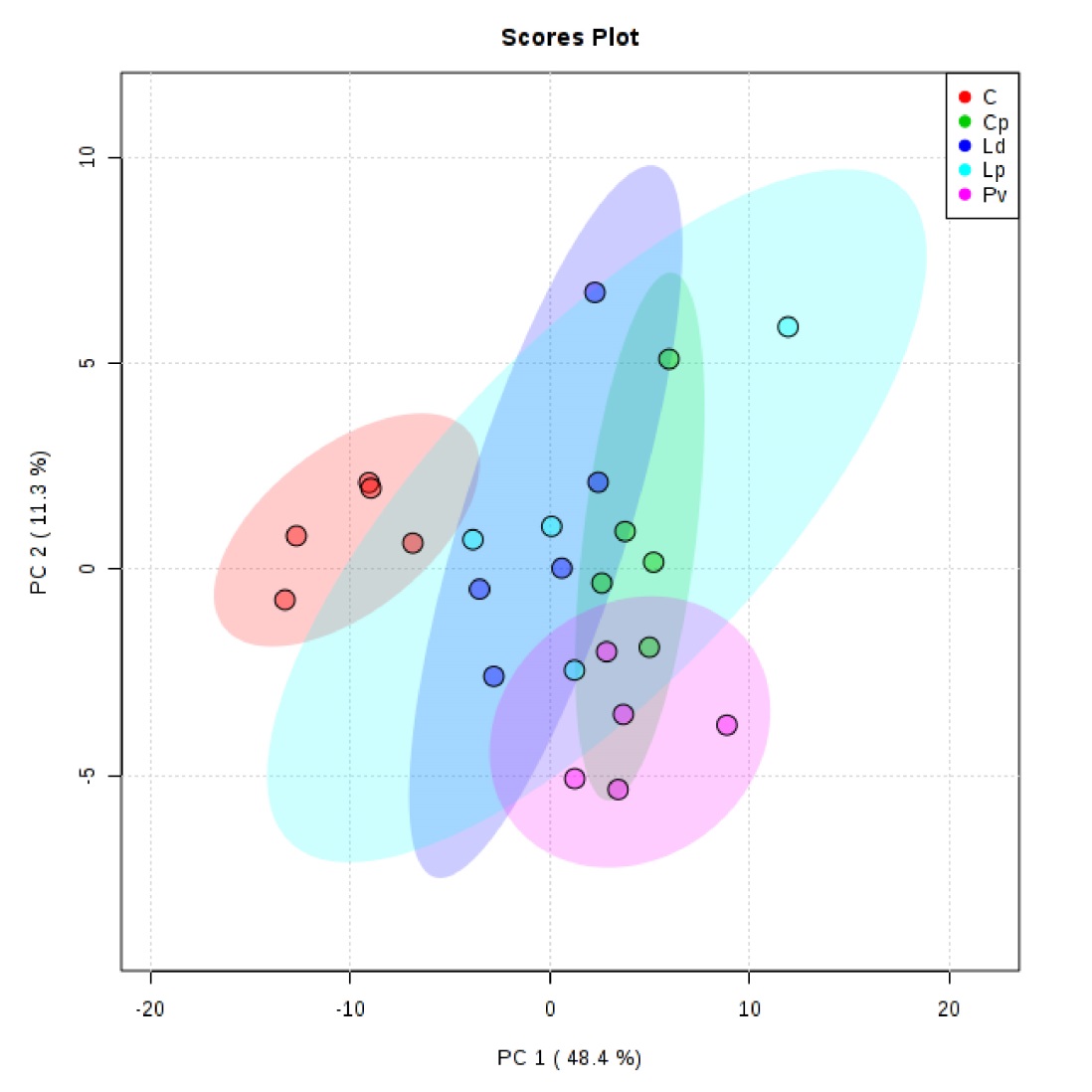


**Fig S3:** Principal component analysis (PCA) of volatile organic compounds (VOCs) in the headspace of damaged black poplar leaves after herbivory by two lepidopteran species, *Lymantria dispar* (Ld = dark blue curcles) and *Laothoe populi* (Lp = light blue circles), or two coleopteran herbivores *Phratora vulgatissima* (Pv = pink circles) and *Chrysomela populi* (Cp = green circles) compared to the headspace of non-damaged control trees (C = red circles).

Before executing the PCA all variables were transformed (using generalized logarithm transformation) and scaled (mean-centered and divided by the standard deviation of each variable). The score plot shows the first two components with the percentage of variance explained enclosed in parentheses. The plots were produced using the metabolomics platform MetaboAnalyst (<https://www.metaboanalyst.ca>)

**Table S1** Relative emission of volatile organic compounds in the headspace of damaged and adjacent undamaged foliage of young *Populus nigra* trees infested by four different herbivore species as compared to the headspace of non-infested leaves of control trees

Samples were collected 44 h after infestation with caterpillars of the two lepidopteran species *Lymantria dispar* and *Laothoe populi*, adults of two coleopteran species, *Phratora vulgatissima* and *Chrysomela populi*, and from untreated control plants. Depicted are the means ± standard errors of the compounds collected via PDMS tubing and measured with GC-MS-TDU.

| **Compound** | **Control (no herbivore)** | | | ***Lymantria dispar*** | | ***Laothoe populi*** | | | | ***Phratora vulgatissima*** | | | | ***Chrysomela populi*** | | |
| --- | --- | --- | --- | --- | --- | --- | --- | --- | --- | --- | --- | --- | --- | --- | --- | --- |
|  |  | | |  | |  | | | |  | | | |  | | |
|  | control for damaged leaves | | control for adj. undamaged leaves | damaged leaves | adjacent undamaged leaves | damaged leaves | | adjacent undamaged leaves | | damaged leaves | | adjacent undamaged leaves | | damaged leaves | | adjacent undamaged leaves |
|  | **mean ± SE** | | **mean ± SE** | **mean ± SE** | **mean ± SE** | **mean ± SE** | | **mean ± SE** | | **mean ± SE** | | **mean ± SE** | | **mean ± SE** | | **mean ± SE** |
| **Monoterpenoids** |  | |  |  |  |  | |  | |  | |  | |  | |  |
| α-Pinene* | 1114 ± 400 | | 3171 ± 568 | 1818 ± 277 | 3802 ± 732 | 4467 ± 1871 | | 2682 ± 440 | | 4000 ± 838 | | 5461 ± 1552 | | 2560 ± 246 | | 7425 ± 2590 |
| Camphene | 354 ± 216 | | 1309 ± 976 | 789 ± 306 | 1678 ± 703 | 307 ± 84 | | 1403 ± 523 | | 1056 ± 885 | | 2273 ± 1309 | | 363 ± 67 | | 1572 ± 1177 |
| 1,8-Cineole* | 1181 ± 343 | | 5905 ± 1498 | 11862 ± 2513 | 5980 ± 1005 | 56382 ± 40537 | | 6707 ± 1400 | | 32718 ± 14983 | | 12712 ± 3676 | | 24888 ± 2847 | | 16499 ± 5555 |
| β-Ocimene* | 217 ± 94 | | 571 ± 167 | 4398 ± 2389 | 995 ± 299 | 7891 ± 4301 | | 1473 ± 983 | | 6259 ± 4063 | | 676 ± 83 | | 7564 ± 2736 | | 1280 ± 636 |
| Linalool* | 377 ± 120 | | 349 ± 98 | 1211 ± 304 | 465 ± 211 | 3736 ± 2926 | | 246 ± 80 | | 2827 ± 1122 | | 364 ± 69 | | 2384 ± 336 | | 9972 ± 9067 |
| Linalool oxide 1* | 277 ± 84 | | 472 ± 81 | 2260 ± 824 | 1175 ± 310 | 2293 ± 1185 | | 2602 ± 1285 | | 3838 ± 2187 | | 3617 ± 1217 | | 3868 ± 1171 | | 3007 ± 609 |
| Linalool oxide 2 | 142 ± 46 | | 217 ± 39 | 613 ± 173 | 279 ± 59 | 1385 ± 668 | | 421 ± 95 | | 709 ± 71 | | 679 ± 105 | | 1157 ± 576 | | 559 ± 100 |
| Linalool oxide 3 | 505 ± 263 | | 1293 ± 335 | 2065 ± 613 | 3481 ± 1870 | 2000 ± 410 | | 2429 ± 563 | | 5003 ± 1487 | | 4662 ± 1643 | | 4209 ± 1222 | | 5137 ± 2014 |
| Camphor* | 10034 ± 2859 | | 25462 ± 5010 | 24642 ± 3516 | 42482 ± 10202 | 64247 ± 32289 | | 30372 ± 2976 | | 83187 ± 15863 | | 53856 ± 12000 | | 50873 ± 6616 | | 71991 ± 29825 |
| Borneol* | 494 ± 258 | | 1222 ± 314 | 1920 ± 534 | 3335 ± 1855 | 1776 ± 369 | | 2198 ± 545 | | 4941 ± 1539 | | 4420 ± 1592 | | 2512 ± 666 | | 4903 ± 1968 |
| Terpinen-4-ol* | 1544 ± 1327 | | 944 ± 294 | 1236 ± 605 | 547 ± 240 | 3117 ± 1034 | | 199 ± 65 | | 1974 ± 1445 | | 743 ± 299 | | 924 ± 126 | | 796 ± 335 |
| α-Terpineol* | 157 ± 32 | | 139 ± 32 | 364 ± 113 | 182 ± 55 | 960 ± 203 | | 177 ± 13 | | 708 ± 193 | | 4197 ± 3921 | | 959 ± 190 | | 326 ± 63 |
| β-Citronellol | 560 ± 140 | | 823 ± 106 | 3545 ± 1430 | 2537 ± 1035 | 6141 ± 3173 | | 2693 ± 1093 | | 9178 ± 2016 | | 4261 ± 1805 | | 9728 ± 3444 | | 3693 ± 1263 |
| Geranyl acetone | 804 ± 169 | | 255 ± 50 | 1124 ± 284 | 422 ± 66 | 18251 ± 17277 | | 757 ± 380 | | 3399 ± 1666 | | 1665 ± 307 | | 2559 ± 967 | | 1183 ± 263 |
| α-Terpinene* | 374 ± 156 | | 1201 ± 265 | 6528 ± 2310 | 2972 ± 1119 | 9366 ± 3825 | | 3615 ± 1256 | | 11532 ± 2854 | | 8207 ± 2374 | | 12698 ± 3703 | | 6221 ± 1525 |
| Unidentified monoterpenoid | 266 ± 143 | | 107 ± 33 | 251 ± 76 | 257 ± 168 | 823 ± 408 | | 86 ± 14 | | 342 ± 82 | | 159 ± 47 | | 1341 ± 332 | | 166 ± 49 |
| **Sesquiterpenoids** |  | |  |  |  |  | |  | |  | |  | |  | |  |
| α-Cubebene ^(a)^ | 86 ± 32 | | 141 ± 41 | 759 ± 177 | 398 ± 121 | 1061 ± 299 | | 511 ± 188 | | 3296 ± 1150 | | 1580 ± 269 | | 2057 ± 258 | | 1154 ± 148 |
| (*E*)-β-Caryophyllene ^(a)^ | 158 ± 51 | | 1078 ± 391 | 1158 ± 409 | 2050 ± 1108 | 1233 ± 485 | | 853 ± 562 | | 1251 ± 541 | | 2663 ± 1323 | | 2321 ± 866 | | 3565 ± 1580 |
| β-Copaene ^(a)^ | 105 ± 29 | | 568 ± 144 | 662 ± 194 | 487 ± 41 | 1013 ± 361 | | 1083 ± 614 | | 2741 ± 1268 | | 1844 ± 335 | | 1917 ± 471 | | 1413 ± 322 |
| α-Humulene* | 387 ± 179 | | 378 ± 61 | 2258 ± 827 | 927 ± 233 | 33720 ± 32005 | | 2007 ± 874 | | 12489 ± 3593 | | 6775 ± 2431 | | 4527 ± 1018 | | 3718 ± 1013 |
| Naphthalene ^(a)^ | 124 ± 46 | | 883 ± 415 | 5410 ± 1924 | 2689 ± 1062 | 253460 ± 251956 | | 17718 ± 14540 | | 7203 ± 3416 | | 10607 ± 3096 | | 14561 ± 10513 | | 6351 ± 2233 |
| (*E,E*)-α-Farnesene ^(a)^ | 4970 ± 4865 | | 811 ± 339 | 1656 ± 486 | 1552 ± 568 | 1051437 ± 1049852 | | 3193 ± 2083 | | 9279 ± 4075 | | 5034 ± 935 | | 3916 ± 816 | | 4632 ± 1230 |
| Nerolidol* | 529 ± 198 | | 614 ± 78 | 1402 ± 584 | 445 ± 121 | 2297 ± 1240 | | 807 ± 258 | | 908 ± 277 | | 410 ± 101 | | 2409 ± 1247 | | 498 ± 86 |
| Guaiol | 75 ± 33 | | 322 ± 51 | 266 ± 70 | 556 ± 91 | 761 ± 519 | | 979 ± 300 | | 547 ± 156 | | 1186 ± 627 | | 523 ± 204 | | 1146 ± 401 |
| τ-Cadinol | 41 ± 4 | | 180 ± 64 | 282 ± 93 | 758 ± 366 | 585 ± 385 | | 386 ± 148 | | 1092 ± 698 | | 1311 ± 991 | | 237 ± 49 | | 904 ± 477 |
| Unidentified sesquiterpenoid 1 | 101 ± 31 | | 356 ± 63 | 2322 ± 694 | 1224 ± 420 | 2012 ± 712 | | 1597 ± 726 | | 10375 ± 4345 | | 4823 ± 947 | | 5121 ± 979 | | 3657 ± 807 |
| Unidentified sesquiterpenoid 2 | 366 ± 82 | | 204 ± 45 | 1540 ± 489 | 790 ± 292 | 1522 ± 612 | | 1148 ± 592 | | 7582 ± 3116 | | 3434 ± 723 | | 3663 ± 893 | | 2600 ± 472 |
| Unidentified sesquiterpenoid 3 | 178 ± 57 | | 2228 ± 2112 | 11825 ± 3296 | 11637 ± 3309 | 26257 ± 17603 | | 15206 ± 6287 | | 70138 ± 29966 | | 49083 ± 9770 | | 24480 ± 8043 | | 36475 ± 7846 |
| Unidentified sesquiterpenoid 4 | 1607 ± 678 | | 8604 ± 2234 | 9313 ± 1183 | 10600 ± 2361 | 17828 ± 10205 | | 13245 ± 3067 | | 48434 ± 22279 | | 33100 ± 7214 | | 21028 ± 3276 | | 24016 ± 4874 |
| Unidentified sesquiterpenoid 5 | 4412 ± 1822 | | 21622 ± 5700 | 22486 ± 2478 | 25962 ± 5800 | 43914 ± 25214 | | 31228 ± 6477 | | 103152 ± 43793 | | 71754 ± 14078 | | 49452 ± 6913 | | 54393 ± 11046 |
| Unidentified sesquiterpenoid 6 | 173 ± 23 | | 465 ± 130 | 2854 ± 896 | 1652 ± 595 | 3560 ± 1022 | | 2947 ± 1685 | | 16799 ± 7467 | | 7454 ± 1432 | | 13697 ± 3104 | | 5838 ± 1266 |
| Unidentified sesquiterpenoid 7 | 139 ± 41 | | 695 ± 109 | 1948 ± 328 | 1588 ± 584 | 4096 ± 1747 | | 2662 ± 1581 | | 14001 ± 5710 | | 9098 ± 1809 | | 6285 ± 2205 | | 5892 ± 1303 |
| Unidentified sesquiterpenoid 8 | 382 ± 253 | | 3059 ± 1203 | 2998 ± 653 | 2416 ± 853 | 56459 ± 54709 | | 5151 ± 2455 | | 5419 ± 1254 | | 5515 ± 1318 | | 15283 ± 10122 | | 5126 ± 1943 |
| Unidentified sesquiterpenoid 9 | 73 ± 15 | | 2822 ± 1123 | 2573 ± 682 | 4456 ± 1158 | 58192 ± 54134 | | 4378 ± 2612 | | 5871 ± 2164 | | 6677 ± 2495 | | 14806 ± 10156 | | 6111 ± 1746 |
| Unidentified sesquiterpenoid 10 | 117 ± 15 | | 1186 ± 534 | 2482 ± 880 | 2338 ± 850 | 252935 ± 250576 | | 4748 ± 3304 | | 13830 ± 6033 | | 7531 ± 1544 | | 5445 ± 1303 | | 7044 ± 1875 |
| Unidentified sesquiterpenoid 11 | 322 ± 70 | | 2750 ± 892 | 6403 ± 1945 | 5554 ± 1741 | 6034 ± 2362 | | 11258 ± 7292 | | 34463 ± 15281 | | 18395 ± 3512 | | 15729 ± 3691 | | 16569 ± 3698 |
| Unidentified sesquiterpenoid 12 | 224 ± 39 | | 2302 ± 1061 | 5364 ± 2241 | 5242 ± 2043 | 5065 ± 2782 | | 10328 ± 7584 | | 28834 ± 17085 | | 15821 ± 3654 | | 10415 ± 5457 | | 14867 ± 4506 |
| Unidentified sesquiterpenoid 13 | 121 ± 27 | | 263 ± 81 | 1215 ± 427 | 535 ± 135 | 1052611 ± 1051907 | | 830 ± 118 | | 1757 ± 644 | | 637 ± 126 | | 1787 ± 541 | | 1287 ± 420 |
| Unidentified sesquiterpenoid 14 | 219 ± 57 | | 231 ± 63 | 565 ± 214 | 483 ± 133 | 577 ± 146 | | 835 ± 497 | | 2180 ± 935 | | 1382 ± 312 | | 1404 ± 426 | | 1109 ± 226 |
| Unidentified sesquiterpenoid 15 | 242 ± 68 | | 273 ± 66 | 324 ± 56 | 471 ± 94 | 534 ± 191 | | 363 ± 151 | | 868 ± 331 | | 770 ± 141 | | 924 ± 410 | | 498 ± 170 |
| **Aromatic compounds** |  | |  |  |  |  | |  | |  | |  | |  | |  |
| Benzaldehyde* | 417 ± 100 | | 194 ± 48 | 1304 ± 684 | 206 ± 34 | 769 ± 252 | | 255 ± 37 | | 336 ± 61 | | 199 ± 62 | | 854 ± 240 | | 850 ± 488 |
| Benzyl alcohol | 552 ± 136 | | 655 ± 153 | 47767 ± 21358 | 1071 ± 287 | 238749 ± 237403 | | 3164 ± 2315 | | 99719 ± 98185 | | 31851 ± 17978 | | 81029 ± 29980 | | 1591 ± 569 |
| Salicylaldehyde* | 292 ± 167 | | 420 ± 348 | 8081 ± 6875 | 102 ± 36 | 1619 ± 1068 | | 156 ± 80 | | 5641 ± 2656 | | 135 ± 20 | | 221698 ± 217627 | | 185 ± 61 |
| 1-Phenylethanone | 601 ± 214 | | 304 ± 104 | 2374 ± 1335 | 282 ± 63 | 1976 ± 1044 | | 301 ± 45 | | 587 ± 111 | | 401 ± 59 | | 1329 ± 787 | | 461 ± 140 |
| 2-Methoxyphenol | 236 ± 59 | | 191 ± 43 | 808 ± 269 | 224 ± 55 | 1418 ± 872 | | 307 ± 107 | | 562 ± 40 | | 207 ± 89 | | 1086 ± 202 | | 463 ± 126 |
| 2-Phenylethanol | 319 ± 155 | | 596 ± 124 | 2103 ± 768 | 612 ± 133 | 5450 ± 3643 | | 725 ± 175 | | 3834 ± 1864 | | 1108 ± 243 | | 3022 ± 519 | | 1870 ± 658 |
| Methyl salicylate | 266 ± 100 | | 4080 ± 2474 | 298 ± 78 | 216 ± 129 | 184 ± 58 | | 89 ± 21 | | 379 ± 81 | | 228 ± 102 | | 1389 ± 440 | | 192 ± 85 |
| Eugenol* | 114 ± 50 | | 46 ± 7 | 16976 ± 16677 | 105 ± 49 | 995 ± 711 | | 150 ± 76 | | 278 ± 153 | | 51 ± 4 | | 22140 ± 20285 | | 215 ± 127 |
| Benzoic acid- *n*-pentyl ester | 139 ± 36 | | 1135 ± 227 | 1516 ± 475 | 1304 ± 230 | 2422 ± 956 | | 1493 ± 383 | | 9501 ± 3839 | | 5103 ± 1013 | | 3492 ± 832 | | 4123 ± 740 |
| 2-Phenylpropan-2-ol | 10034 ± 2859 | | 25462 ± 5010 | 24642 ± 3516 | 42482 ± 10202 | 64247 ± 32289 | | 30372 ± 2976 | | 83187 ± 15863 | | 53856 ± 12000 | | 50873 ± 6616 | | 71991 ± 29825 |
|  |  | |  |  |  |  | |  | |  | |  | |  | |  |
| **Compound** | | **Control (no herbivore)** | | ***Lymantria dispar*** | | ***Laothoe populi*** | | | ***Phratora vulgatissima*** | | | | ***Chrysomela populi*** | | | |
|  |  |  | |  | |  | | |  | | | |  | | | |
|  |  | control for damaged leaves | control for adj. undamaged leaves | damaged leaves | adjacent undamaged leaves | damaged leaves | adjacent undamaged leaves | | damaged leaves | | adjacent undamaged leaves | | damaged leaves | | adjacent undamaged leaves | |
|  |  | **mean ± SE** | **mean ± SE** | **mean ± SE** | **mean ± SE** | **mean ± SE** | **mean ± SE** | | **mean ± SE** | | **mean ± SE** | | **mean ± SE** | | **mean ± SE** | |
| **Nitrogenous compounds** | |  |  |  |  |  |  | |  | |  | |  | |  | |
| 2-Methylbutyraldoxime* | | 773 ± 618 | 456 ± 185 | 17406 ± 10139 | 595 ± 232 | 22099 ± 14042 | 469 ± 80 | | 24609 ± 4231 | | 255 ± 36 | | 34642 ± 7792 | | 1945 ± 1428 | |
| 3-Methylbutyraldoxime* | | 2283 ± 526 | 692 ± 272 | 4222 ± 1156 | 679 ± 173 | 3014 ± 1115 | 748 ± 306 | | 914 ± 356 | | 563 ± 317 | | 1789 ± 515 | | 1094 ± 255 | |
| Methoxy phenyloxime | | 52885 ± 32679 | 27546 ± 13920 | 63928 ± 32245 | 32211 ± 12947 | 210029 ± 111149 | 47280 ± 15113 | | 63393 ± 33543 | | 32296 ± 15592 | | 75678 ± 30192 | | 25813 ± 14517 | |
| 1-Methyl-2-pyrrolidinone | | 204 ± 39 | 2242 ± 2171 | 1472 ± 424 | 4339 ± 2597 | 1136 ± 703 | 747 ± 426 | | 422 ± 53 | | 70 ± 16 | | 2983 ± 1340 | | 4358 ± 2779 | |
| Benzyl-cyanide* | | 6002 ± 5897 | 19627 ± 12486 | 167202 ± 162887 | 2594 ± 1879 | 59559 ± 25678 | 12000 ± 6077 | | 288693 ± 98010 | | 1529 ± 1324 | | 586122 ± 196588 | | 10521 ± 10343 | |
| 2-phenylnitroethane* | | 574 ± 96 | 387 ± 70 | 1277 ± 640 | 556 ± 241 | 1980 ± 1320 | 343 ± 57 | | 472 ± 110 | | 266 ± 47 | | 1421 ± 439 | | 326 ± 133 | |
| N-phenylaniline | | 307 ± 75 | 41 ± 16 | 634 ± 387 | 67 ± 20 | 285 ± 113 | 100 ± 33 | | 177 ± 63 | | 35 ± 11 | | 257 ± 120 | | 76 ± 16 | |
| Unidentified nitrogenous | | 1331 ± 224 | 1135 ± 487 | 10102 ± 4760 | 5741 ± 3590 | 12372 ± 7265 | 3945 ± 1728 | | 7406 ± 3527 | | 2779 ± 2347 | | 80405 ± 58354 | | 6224 ± 3498 | |
| Indole* | | 173 ± 35 | 74 ± 33 | 1148 ± 929 | 106 ± 32 | 564 ± 288 | 69 ± 9 | | 20286 ± 3785 | | 70 ± 10 | | 24022 ± 7726 | | 481 ± 364 | |
| **Green Leaf Volatiles** | |  |  |  |  |  |  | |  | |  | |  | |  | |
| Hexan-1-ol | | 906 ± 182 | 500 ± 285 | 2837 ± 1496 | 670 ± 204 | 976 ± 242 | 400 ± 111 | | 803 ± 293 | | 275 ± 82 | | 2497 ± 1020 | | 755 ± 363 | |
| 2-Hexenal | | 15932 ± 13856 | 421 ± 110 | 6657 ± 3251 | 360 ± 106 | 27762 ± 22810 | 717 ± 459 | | 4523 ± 969 | | 233 ± 45 | | 7663 ± 2305 | | 4897 ± 4238 | |
| 3-Hexenal | | 1042 ± 396 | 104 ± 33 | 3058 ± 1477 | 160 ± 65 | 50561 ± 49689 | 139 ± 62 | | 1403 ± 687 | | 101 ± 57 | | 14918 ± 8341 | | 255 ± 158 | |
| 3-Hexenol* | | 17845 ± 15507 | 165 ± 77 | 61508 ± 50904 | 521 ± 242 | 72640 ± 43962 | 542 ± 205 | | 5095 ± 3313 | | 334 ± 163 | | 21429 ± 12880 | | 410 ± 66 | |
| 2-Hexenol | | 1105 ± 404 | 804 ± 427 | 2476 ± 1002 | 263 ± 124 | 7555 ± 4196 | 506 ± 196 | | 2916 ± 1039 | | 981 ± 329 | | 8662 ± 2083 | | 952 ± 602 | |
| 3-Hexenyl acetate* | | 4052 ± 1274 | 7605 ± 2849 | 7737 ± 580 | 13022 ± 3006 | 9875 ± 2925 | 11934 ± 2499 | | 14508 ± 2035 | | 17341 ± 2686 | | 7310 ± 1633 | | 31251 ± 12879 | |
| Hexyl acetate | | 2233 ± 417 | 3089 ± 587 | 6748 ± 2208 | 4420 ± 895 | 4920 ± 828 | 3873 ± 863 | | 5338 ± 712 | | 5227 ± 736 | | 6009 ± 1605 | | 7111 ± 1571 | |
| 2-Hexenyl acetate* | | 2187 ± 1105 | 471 ± 101 | 3062 ± 725 | 497 ± 126 | 3900 ± 2278 | 657 ± 162 | | 1734 ± 496 | | 742 ± 136 | | 5548 ± 2705 | | 1186 ± 422 | |
| 3-Hexenyl isobutyrate* | | 1618 ± 451 | 3329 ± 630 | 4476 ± 1112 | 5967 ± 1449 | 7754 ± 3850 | 4002 ± 383 | | 10362 ± 1966 | | 6891 ± 1493 | | 5603 ± 919 | | 9252 ± 3781 | |
| 3-Hexenyl butyrate | | 1739 ± 369 | 1075 ± 376 | 4742 ± 2902 | 1436 ± 526 | 4838 ± 3133 | 1650 ± 639 | | 2191 ± 433 | | 863 ± 345 | | 5489 ± 2120 | | 1857 ± 1018 | |
| 3-Hexenyl benzoate* | | 1221 ± 400 | 2586 ± 894 | 3066 ± 554 | 4287 ± 1575 | 6891 ± 4794 | 6158 ± 3075 | | 3583 ± 1032 | | 11774 ± 7296 | | 3240 ± 585 | | 7857 ± 4423 | |
| 3-Hexenyl 2-methyl butanoate | | 114 ± 21 | 192 ± 36 | 1064 ± 411 | 507 ± 183 | 1896 ± 1200 | 539 ± 177 | | 2199 ± 522 | | 853 ± 336 | | 2058 ± 503 | | 817 ± 262 | |
| **Other** | |  |  |  |  |  |  | |  | |  | |  | |  | |
| Heptane | | 133820 ± 40375 | 92514 ± 16179 | 448936 ± 149269 | 276418 ± 198043 | 378177 ± 175996 | 33453 ± 16577 | | 149208 ± 47230 | | 52284 ± 17519 | | 374848 ± 76138 | | 2839497 ± 2774479 | |
| Oxabicyclo-hexan-2-one | | 142 ± 16 | 177 ± 103 | 616 ± 171 | 98 ± 24 | 974 ± 551 | 118 ± 18 | | 340 ± 70 | | 71 ± 22 | | 337 ± 125 | | 201 ± 49 | |
| Hydroxyethoxyethanol | | 2471 ± 472 | 807 ± 290 | 3528 ± 1215 | 2021 ± 718 | 4959 ± 2023 | 1168 ± 212 | | 2527 ± 371 | | 641 ± 144 | | 3994 ± 750 | | 629 ± 117 | |
| Methylheptenone | | 504 ± 323 | 92 ± 20 | 481 ± 247 | 164 ± 59 | 4820 ± 4594 | 115 ± 23 | | 143 ± 22 | | 92 ± 7 | | 457 ± 153 | | 262 ± 106 | |
| 2-Hydroxycyclohexanone | | 1157 ± 172 | 852 ± 302 | 1384 ± 366 | 1271 ± 588 | 1316 ± 745 | 975 ± 301 | | 475 ± 192 | | 1257 ± 446 | | 2420 ± 1588 | | 878 ± 489 | |
| 1.2-Cyclohexanediol | | 226 ± 51 | 208 ± 66 | 1435 ± 711 | 384 ± 125 | 15339 ± 10592 | 606 ± 388 | | 4430 ± 1363 | | 140 ± 74 | | 5685 ± 3360 | | 784 ± 235 | |
| Nonanal* | | 4088 ± 2373 | 2494 ± 1728 | 15045 ± 11384 | 3067 ± 1905 | 3500 ± 2529 | 1368 ± 631 | | 1482 ± 895 | | 178 ± 33 | | 2674 ± 1259 | | 4434 ± 3103 | |
| Decanal | | 3463 ± 1625 | 2647 ± 1067 | 7432 ± 2709 | 2861 ± 1689 | 336133 ± 314834 | 1399 ± 621 | | 19393 ± 13568 | | 427 ± 193 | | 61014 ± 25721 | | 1598 ± 794 | |
| β-Cyclocitral | | 933 ± 204 | 537 ± 271 | 1451 ± 518 | 634 ± 238 | 995 ± 389 | 379 ± 179 | | 247 ± 59 | | 355 ± 144 | | 748 ± 335 | | 1068 ± 436 | |
| Nonanoic acid | | 8919 ± 1065 | 3336 ± 1677 | 23322 ± 9515 | 3336 ± 1202 | 11011 ± 2385 | 2774 ± 1578 | | 7092 ± 2692 | | 981 ± 533 | | 15462 ± 9147 | | 4267 ± 2395 | |
| Undecanal | | 288 ± 57 | 203 ± 48 | 2260 ± 1468 | 514 ± 180 | 1122 ± 550 | 195 ± 56 | | 546 ± 227 | | 177 ± 24 | | 1033 ± 232 | | 599 ± 250 | |
| Octylether | | 942 ± 135 | 441 ± 133 | 1428 ± 603 | 759 ± 148 | 10253 ± 8101 | 625 ± 171 | | 1387 ± 276 | | 972 ± 446 | | 3908 ± 1038 | | 1043 ± 346 | |
| Jasmone | | 5317 ± 1805 | 29915 ± 7504 | 30665 ± 3306 | 35189 ± 7598 | 59918 ± 34714 | 41967 ± 8444 | | 130653 ± 53135 | | 92114 ± 17122 | | 65349 ± 9043 | | 70956 ± 14125 | |

* Confirmed by comparison of retention time and mass spectrum to that of internal standard (source of standard shown in table S2).

(a) compounds were identified by ≥ 95% similarity to structures in WILEY8 or NIST databases.

**Table S2** Internal standards used for the identification of volatile organic compounds in black poplar

| **Standard** | **CAS-Nr.** | **Source** |
| --- | --- | --- |
| (*E*)-2-Hexenyl acetate | 2497-18-9 | Sigma-Aldrich (Taufkirchen, Germany) |
| (*Z*)-3-Hexen-1-ol | 928-96-1 | Bedoukian (Danbury, CT, USA) |
| (*Z*)-3-Hexenyl benzoate | 3681-71-8 | Sigma-Aldrich (Taufkirchen, Germany) |
| (*Z*)-3-Hexenyl acteate | 3681-71-8 | Sigma-Aldrich (Taufkirchen, Germany) |
| (*Z*)-3-Hexenyl isobutyrate | 41519-23-7 | Sigma-Aldrich (Taufkirchen, Germany) |
| 2-Methylbutyraldoxime (*E:Z*, 3:1) | 49805-56-3 | Chemical synthesis (Irmisch *et al*. (2013) |
| 2-Phenylnitroethane | 6125-24-2 | Apin Chemicals (Abingdon, UK) |
| 3-Methylbutyraldoxime (*E:Z*, 2:1) | 626-90-4 | Chemical synthesis (Irmisch *et al*. (2013) |
| Benzaldehyde | 100-52-7 | Fluka (Munich, Germany) |
| Benzyl cyanide | 140-29-4 | Sigma-Aldrich (Taufkirchen, Germany) |
| Borneol | 464-45-9 | Fluka (Munich, Germany) |
| Camphor | 24368-68-3 | Fluka (Munich, Germany) |
| 1,8-Cineole | 470-82-6 | Sigma-Aldrich (Taufkirchen, Germany) |
| Eugenol | 97-53-0 | Sigma-Aldrich (Taufkirchen, Germany) |
| Indole | 120-72-9 | Sigma-Aldrich (Taufkirchen, Germany) |
| Linalool | 78-70-6 | Sigma-Aldrich (Taufkirchen, Germany) |
| Linalool oxide | 60047-17-8 | Fluka (Munich, Germany) |
| Nerolidol | 7212-44-4 | Sigma-Aldrich (Taufkirchen, Germany) |
| Nonanal | 124-19-6 | Sigma-Aldrich (Taufkirchen, Germany) |
| Salicylaldehyde | 90-02-8 | Acros Organics (Thermo Fischer Scientific, Geel, Belgium) |
| Terpinen-4-ol | 562-74-3 | Thermo Fischer Scientific (Geel, Belgium) |
| α-Humulene | 6753-98-6 | Fluka (Munich, Germany) |
| α-pinene | 7785-26-4 | Fluka (Munich, Germany) |
| α-terpinene | 99-86-5 | Fluka (Munich, Germany) |
| α-Terpineol | 10482-56-1 | Sigma-Aldrich (Taufkirchen, Germany) |
| (*E*)-ß-ocimene | 3338-55-4 | Chemos (Regenstauf, Germany) |

# References

Irmisch S et al. (2013) Two herbivore-induced cytochrome P450 enzymes CYP79d6 and CYP79d7 catalyze the formation of volatile aldoximes involved in poplar defense. Plant Cell 25:4737-4754.doi:10.1105/tpc.113.118265
